# Supplementary material for: Proteome Dynamics During Transition From Exponential to Stationary Phase Under Aerobic and Anaerobic Conditions in Yeast
Source: Mol Cell Proteomics. 2023 Apr 17;22(6):100552. doi: 10.1016/j.mcpro.2023.100552 (PMC10227427; doi:10.1016/j.mcpro.2023.100552)
Supplement: Supplementary Data 1 [file mmc1.pdf]

Additional information to reviewer access for mass spectrometric raw data:

Proteome dynamics during transition from exponential to stationary phase under aerobic and anaerobic conditions in yeast

Maxime den Ridder, Wiebeke van den Brandeler, Meryem Altiner, Pascale Daran-Lapujade* and Martin Pabst*
Delft University of Technology, Department of Biotechnology, van der Maasweg 9, 2629 HZ Delft, The Netherlands
*Contacts: P.A.S.Daran-Lapujade@tudelft.nl or m.pabst@tudelft.nl

A. Access to raw data via ProteomeXchange server:

Reviewer account details:	https://www.ebi.ac.uk/pride/login
Project accession:	PXD031412
Username:	reviewer_pxd031412@ebi.ac.uk
Password:	LcsErtel
