# Supplementary material for: Proteome Dynamics During Transition From Exponential to Stationary Phase Under Aerobic and Anaerobic Conditions in Yeast
Source: Mol Cell Proteomics. 2023 Apr 17;22(6):100552. doi: 10.1016/j.mcpro.2023.100552 (PMC10227427; doi:10.1016/j.mcpro.2023.100552)
Supplement: Supplementary Data 2 [file mmc2.pdf]

Supplementary information material to:

Proteome dynamics during transition from exponential to stationary phase under aerobic and anaerobic conditions in yeast

Maxime den Ridder, Wiebeke van den Brandeler, Meryem Altiner, Pascale Daran-Lapujade* and Martin Pabst*
Delft University of Technology, Department of Biotechnology, van der Maasweg 9, 2629 HZ Delft, The Netherlands
*Contacts: P.A.S.Daran-Lapujade@tudelft.nl or m.pabst@tudelft.nl

TABLE OF CONTENTS

SI Table 1: Biomass-specific substrate consumption and product formation rates — Page 2
SI Table 2: Statistical power calculations — Page 2
SI Figure 1: Upset plots showing the shared proteins and peptides across experiments — Page 3
SI Figure 2: Estimated absolute abundance of detected proteins — Page 3
SI Figure 3: Hierarchical clustering of growth phase time points of different yeast strains and conditions — Page 4
SI Figure 4: Statistical power calculations — Page 11
SI Figure 5: Experimental variation and statistical power for performed TMT experiments — Page 12
SI Figure 6: Profiles of proteins involved in glycerol, glycogen and trehalose metabolism — Page 13
SI Figure 7: Volcano plots global proteome changes between aerobic and anaerobic conditions — Page 14
SI Figure 8: Protein profile changes of heat shock proteins — Page 15
SI Figure 9: Protein profile changes of selected pathways — Page 16

SI Table 1. Biomass-specific substrate consumption and product formation rates of MG (IMX372) and control (CEN.PK113-7D) strain during aerobic and anaerobic exponential growth. The average and standard deviations were calculated for three biological replicates per strain. The biomass specific consumption or formation rates were calculated for the substrate glucose (q_s), ethanol (q_{EtOH}), glycerol(q_{Glyc}), acetate (q_{Ace}), carbon dioxide (q_{CO2}), and oxygen (q_{O2}). Significant differences ($p<0.01$) between control and MG yeast were calculated with an two-sided two-sample unpaired t-test and are highlighted with an asterisk. #The qCO_2 of IMX372 under anaerobic conditions is likely underestimated due to technical complications in the CO_2 off gas analysis equipment.

	AEROBIC				ANAEROBIC			
	CEN.PK113-7D		IMX372		CEN.PK113-7D		IMX372	
	Average	Stdev	Average	Stdev	Average	Stdev	Average	Stdev
μ^{max} (h^{-1})	0,413	0,012	0,396	0,002	0,366	0,003	0,355	0,012
q_s ($mmol \cdot gDW^{-1} \cdot h^{-1}$)	-18,890	1,029	-18,585	0,580	-23,264	1,792	-20,677	0,659
q_{EtOH} ($mmol \cdot gDW^{-1} \cdot h^{-1}$)	30,166	1,999	27,063	0,446	31,814	3,049	30,999	1,677
q_{Glyc} ($mmol \cdot gDW^{-1} \cdot h^{-1}$)	1,736	0,074	1,518	0,147	4,607	0,395	4,297	0,198
q_{Ace} ($mmol \cdot gDW^{-1} \cdot h^{-1}$)	0,494	0,082	0,493	0,080	0,560	0,092	0,540	0,045
q_{CO2} ($mmol \cdot gDW^{-1} \cdot h^{-1}$)	33,024	2,056	28,175	0,912	32,037	1,213	27,053*#	1,343
q_{O2} ($mmol \cdot gDW^{-1} \cdot h^{-1}$)	9,479	0,533	7,978	0,149				
RQ	3,517	0,173	3,574	0,215				
$Y^{biomass/glucose}$ ($gDW \cdot gglucose^{-1}$)	-0,022	0,001	-0,021	0,001	-0,016	0,001	-0,017	0,000
$Y^{ethanol/glucose}$ ($mol \cdot mol^{-1}$)	1,598	0,111	1,458	0,025	1,379	0,032	1,503	0,129
$Y^{glycerol/glucose}$ ($mol \cdot mol^{-1}$)	0,092	0,009	0,082	0,009	0,199	0,027	0,208	0,004
$Y^{acetate/glucose}$ ($mol \cdot mol^{-1}$)	0,026	0,003	0,026	0,004	0,024	0,004	0,026	0,001
Carbon recovery	1,000	0,000	1,000	0,000	1,000	0,000	1,000	0,000

SI Table 2. Determination of required number of replicates for different experimental variations. For different experimental variations (in the table expressed as relative standard deviations (RSD), %) and different fold change levels (1.25, 1.5, 2, 2.5 and 3) the required number of biological replicates was determined. For lower RSD values (5, 10 and 15%) a 1.5 fold change, and for larger RSD values (20 and 30%) at least 3 biological replicates are required. Green boxes show RSD/fold change combinations that require 3 biological replicates, whereas the grey boxes show RSD/fold change combinations that require more than 3 biological replicates. The power calculations were performed using the python "statsmodels module" (https://www.statsmodels.org, stats.power.TTestIndPower.solve_power(), using alpha=0.05 and power of 0.8). Boxes in green show RSD/fold change combinations that can be measured with 3 biological replicates, grey boxes show RSD/fold change combinations that require more than 3 biological replicates (as indicated by the number in the boxes).

Fold change	5% RSD	10% RSD	15% RSD	20% RSD	30% RSD	40% RSD
1.25	2.1	3.8	6.8	11.1	23.6	41.2
1.5	2.0	2.1	2.8	3.8	6.8	11.1
2	2.0	2.0	2.0	2.1	2.8	3.8
2.5	2.0	2.0	2.0	2.0	2.1	2.5
3	2.0	2.0	2.0	2.0	2.0	2.1

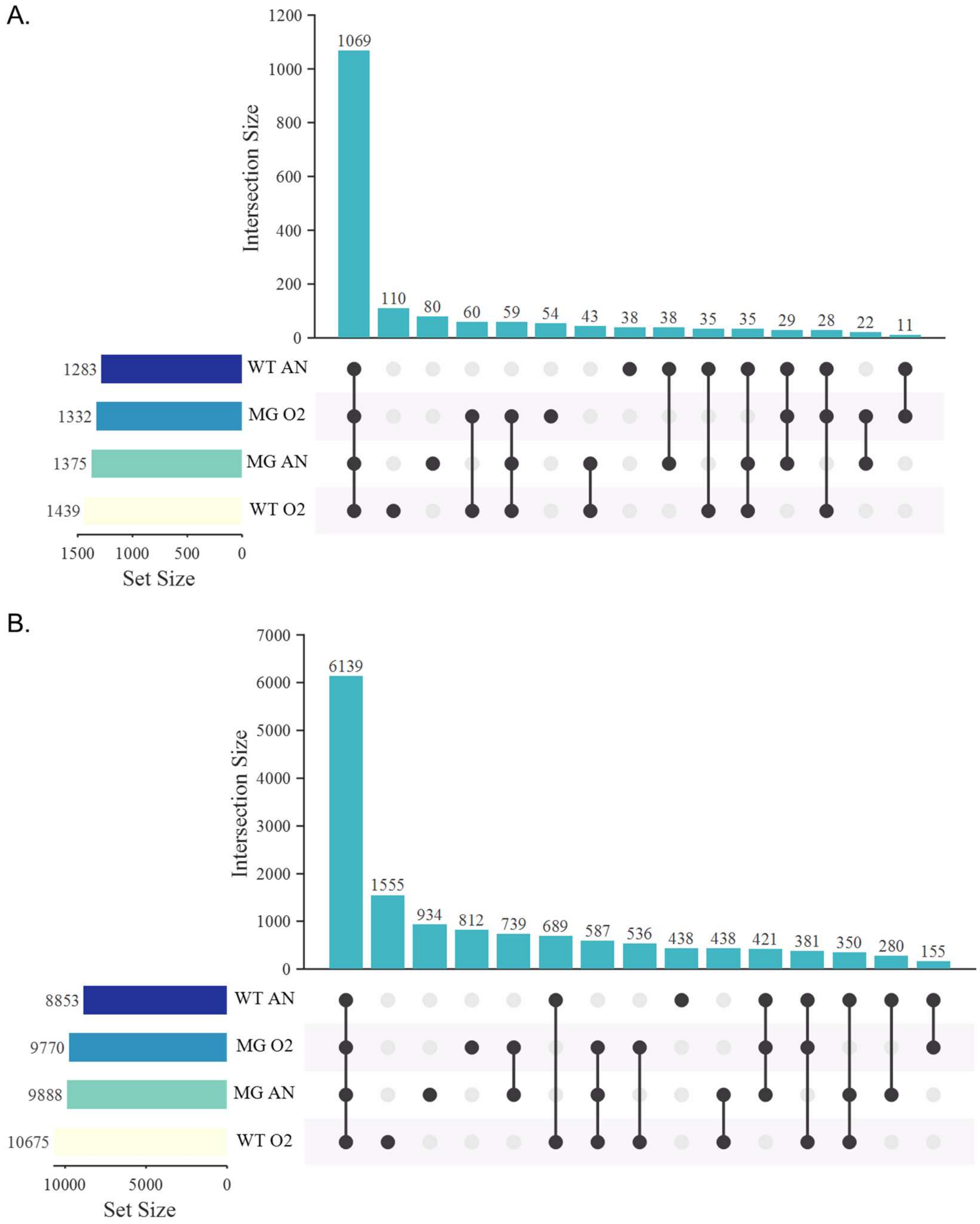

SI Figure 1. UpSet plots visualizing the number of identified proteins (A) and unique peptides (B) that are unique to each condition of shared between the conditions. Horizontal bars indicate the number of proteins or unique peptides that were identified in each condition (WT AN = CEN.PK113-7D anaerobic, MG O2 = IMX372 aerobic, MG AN = IMX372 anaerobic and WT O2 = CEN.PK113-7D aerobic). The nature of each intersection is indicated by the dots below the vertical bars. The number of shared identified proteins (A) and unique peptides (B) are illustrated with the vertical bars.

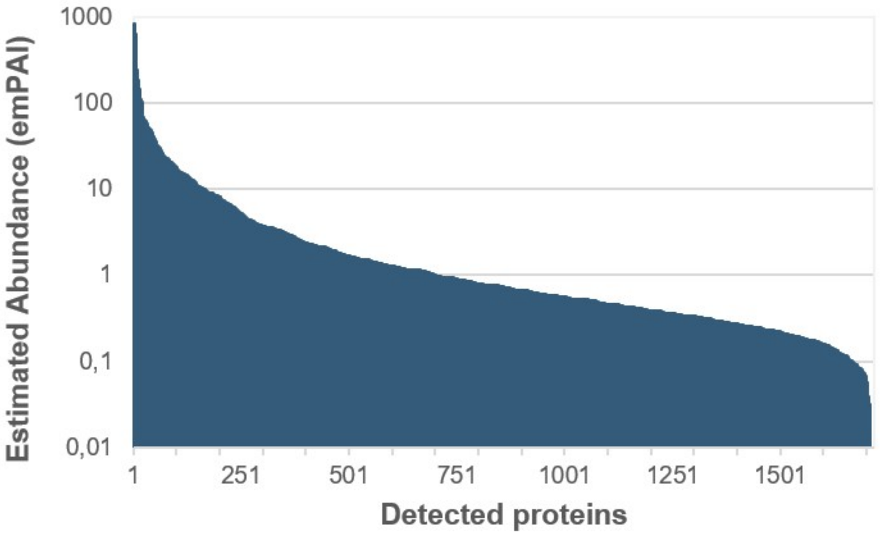

SI Figure 2. Estimated absolute abundance of detected proteins across all experiments. The absolute abundance was estimate using the Exponentially Modified Protein Abundance Index (emPAI) for all identified proteins across all experiments. The absolute protein amount is estimated by the number of sequenced peptides per protein. The abundance of each protein is an average of all experiments and detected in at least one biological replicate.

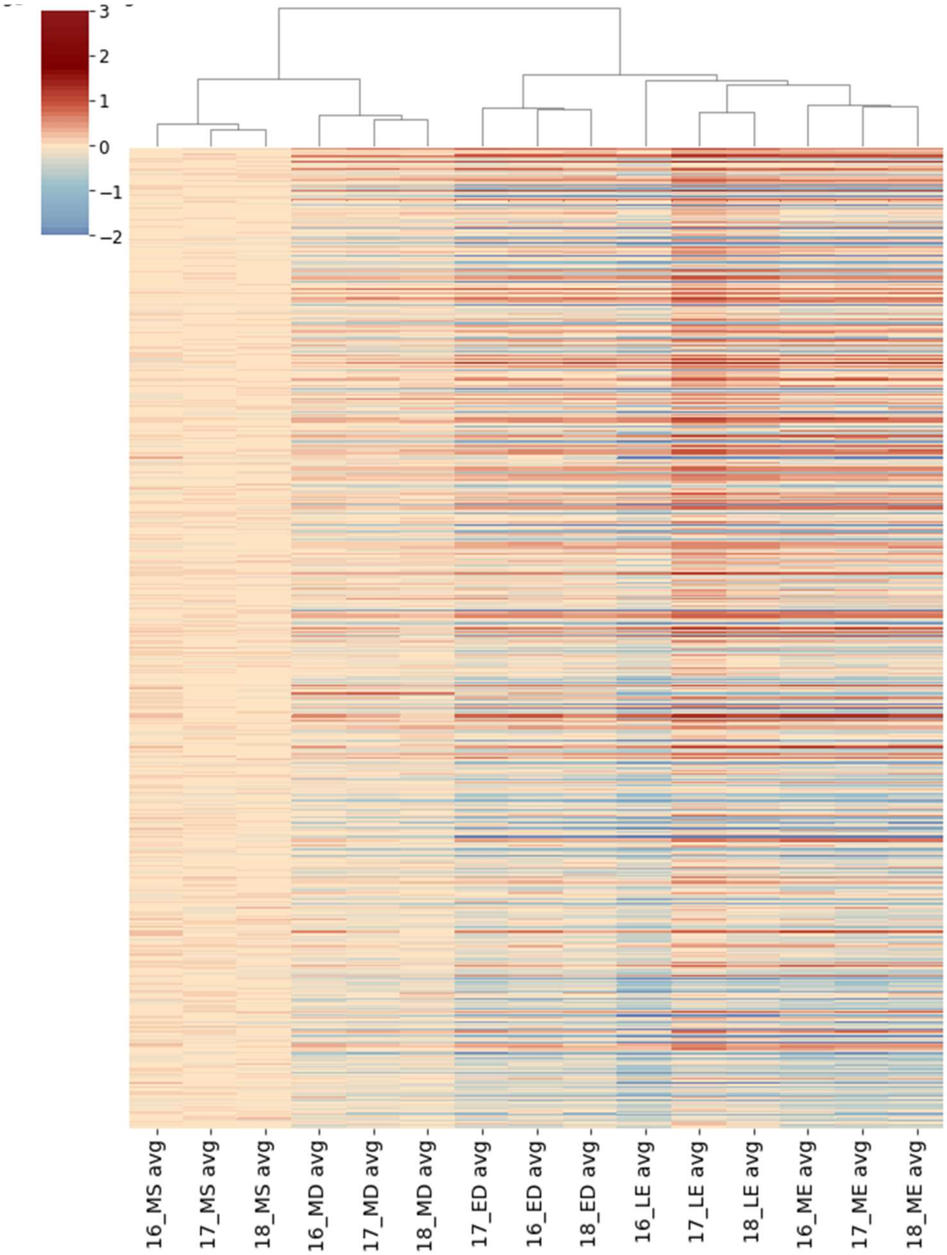

SI Figure 3.1. **Cluster analysis of replicates and growth phase time points from minimal glycolysis (MG) yeast grown under aerobic (O2) conditions.** Cluster analysis was conducted using the average linkage method and Euclidean distances. The log2 of the ratio to the bridging sample was calculated and visualized such that a ratio of 1 is colored white, a negative ratio is colored blue, and a positive ratio is colored red. The labels of the columns represent the reactor number (which indicates the biological replicate) and the sampling time point (which represents the growth phase): ME (mid-exponential), LE (late-exponential), ED (early-diauxic), MD (mid-diauxic shift), ES (early-stationary), and MS (mid-stationary). Generally, the biological replicates of the individual growth phase samples showed a clear grouping.

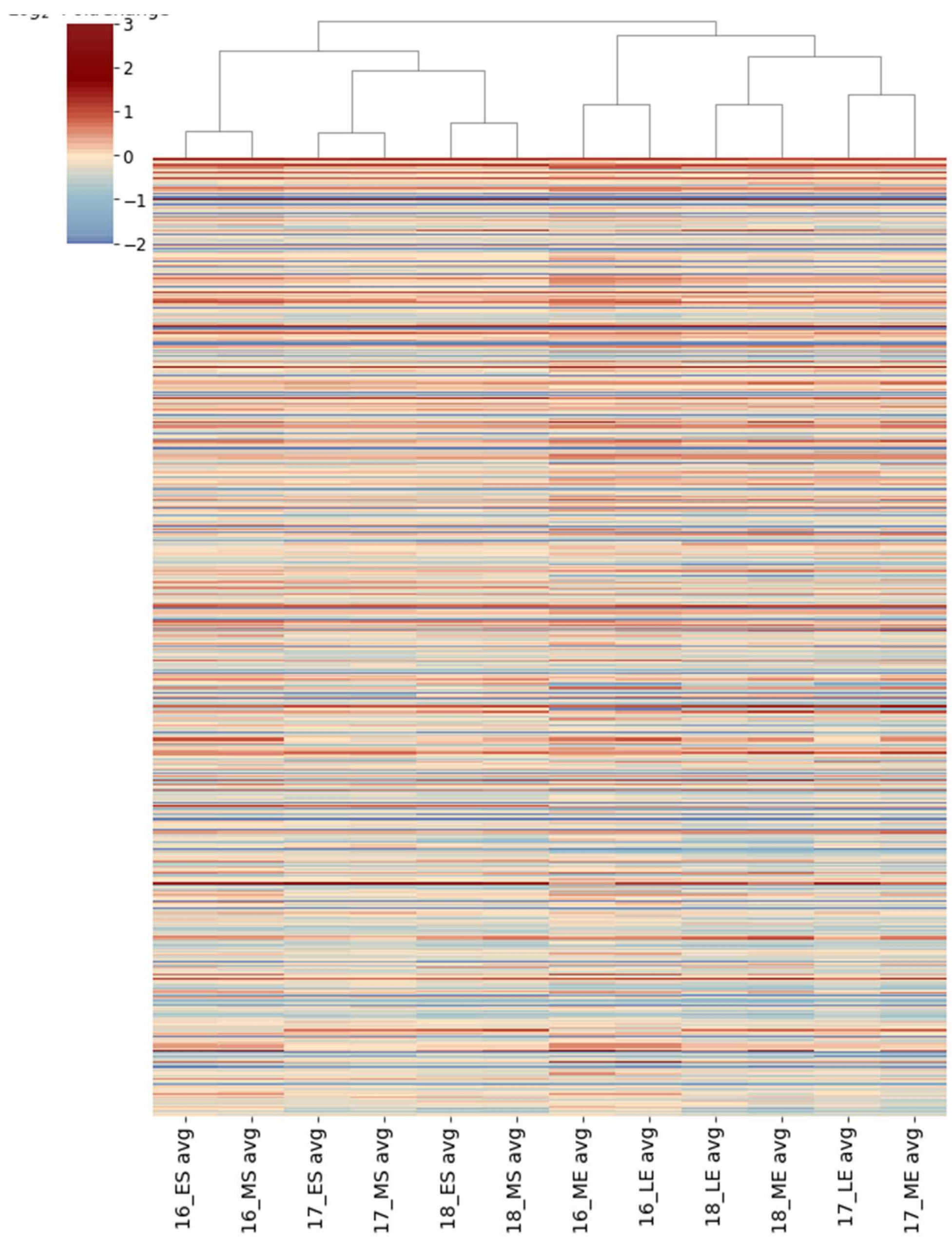

SI Figure 3.2. Cluster analysis of replicates and growth phase time points from minimal glycolysis (MG) yeast grown under anaerobic (AN) conditions. The individual time points from the biological replicates were clustered using the Euclidean distance and average linkage method. The log2 of the ratio to the bridging sample was plotted to show a ratio of 1 in white, negative ratios in blue, and positive ratios in red. The column labels represent the reactor number (indicating the biological replicate) and the sampling time point (denoting the growth phase): ME (mid-exponential), LE (late-exponential), ES (early-stationary) and MS (mid-stationary). Overall, the biological replicates from ES and MS as well as ME and LE grouped together, which is expected given the minimal proteome differences between these growth phases. A more pronounced difference was observed between exponential and stationary growth phases.

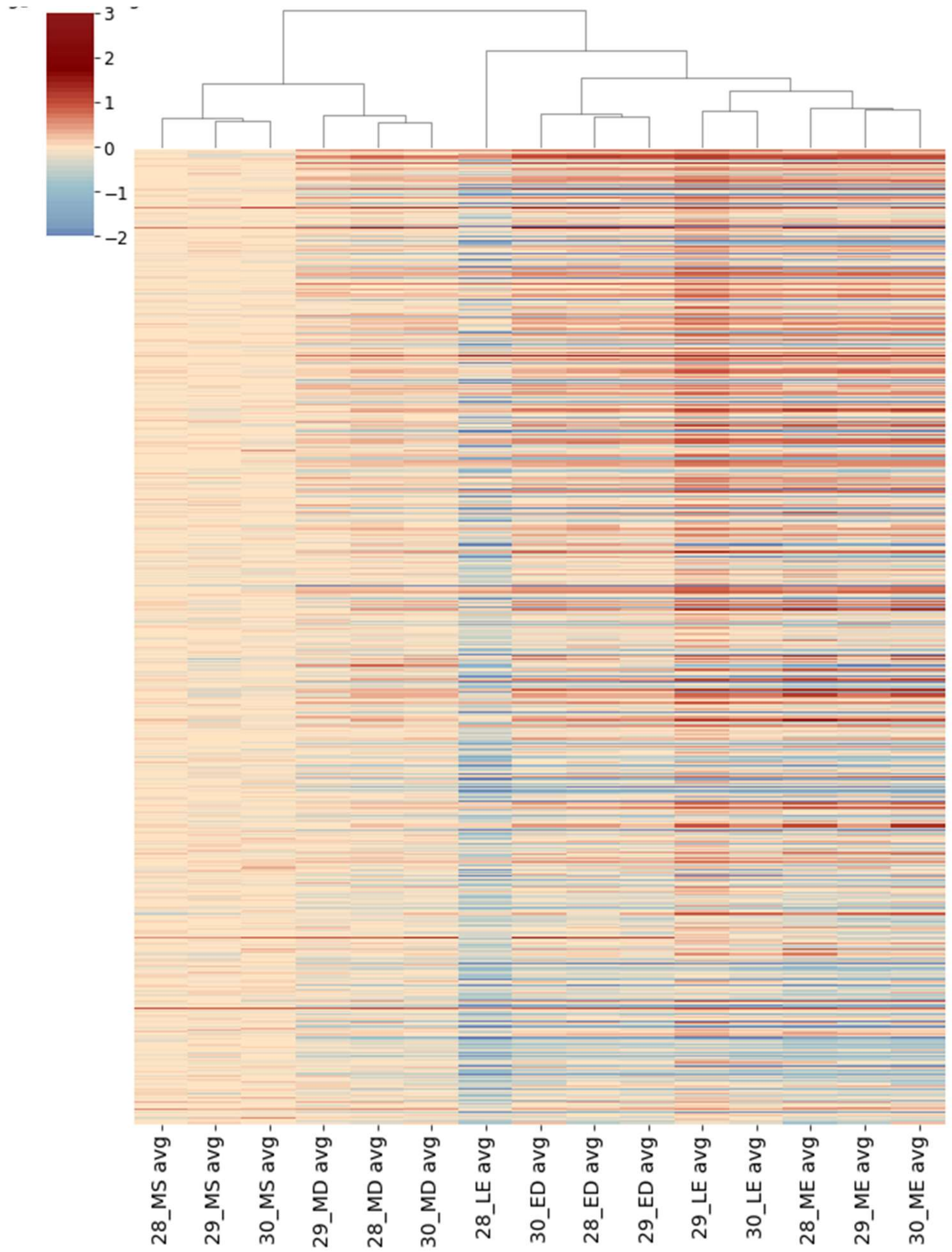

SI Figure 3.3. **Cluster analysis of replicates and growth phase time points from wild-type (WT) yeast grown under aerobic (O2) conditions.** Cluster analysis was conducted using the average linkage method and Euclidean distances. The log2 of the ratio to the bridging sample was calculated and visualized such that a ratio of 1 is colored white, a negative ratio is colored blue, and a positive ratio is colored red. The labels of the columns represent the reactor number (which indicates the biological replicate) and the sampling time point (which represents the growth phase): ME (mid-exponential), LE (late-exponential), ED (early-diauxic), MD (mid-diauxic shift), ES (early-stationary), and MS (mid-stationary). Generally, the time points of the individual biological replicates showed a clear grouping, except for a slight variation between the LE replicates.

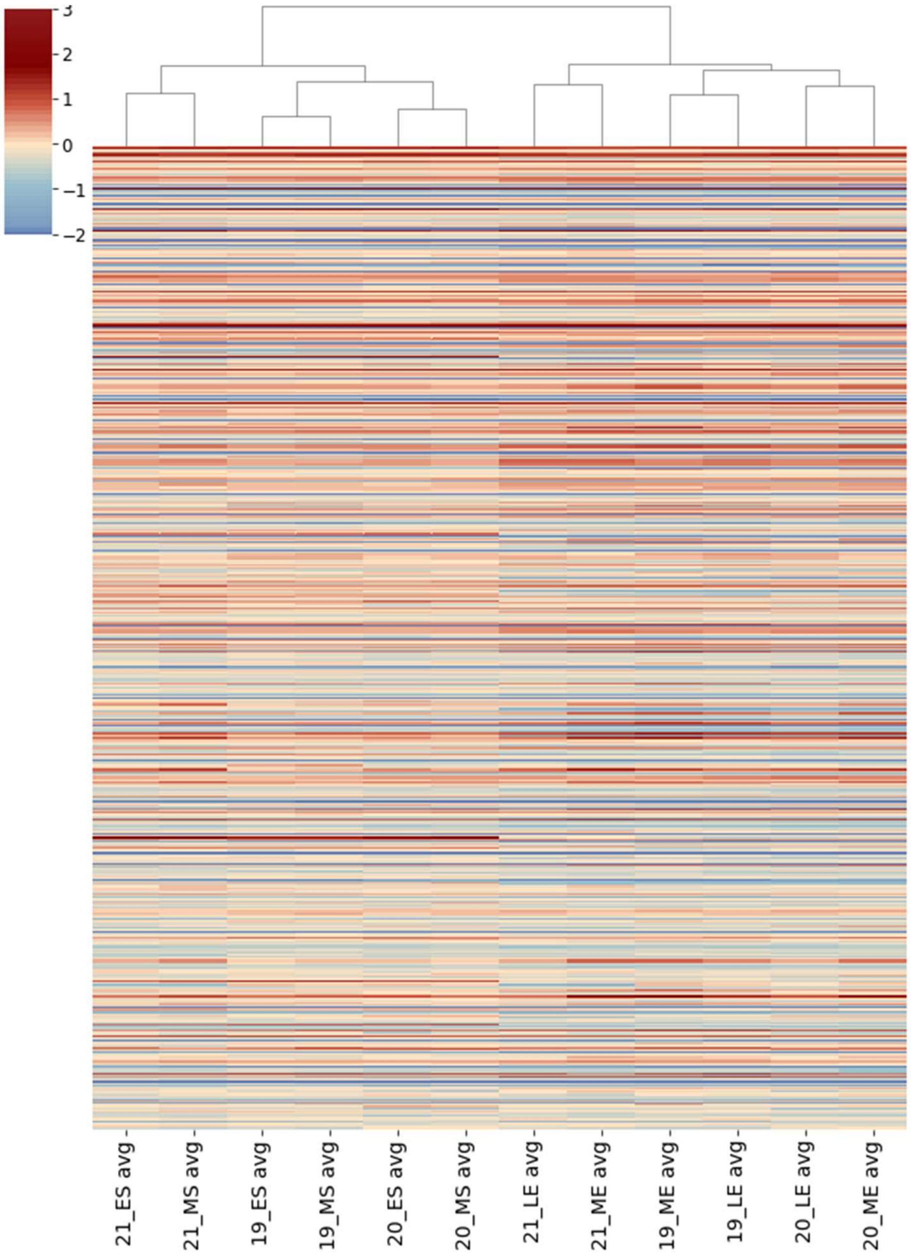

SI Figure 3.4. Cluster analysis of replicates and growth phase time points from wild-type (WT) yeast grown under anaerobic (AN) conditions. The individual time points from the biological replicates were clustered using the Euclidean distance and average linkage method. The log2 of the ratio to the bridging sample was plotted to show a ratio of 1 in white, negative ratios in blue, and positive ratios in red. The column labels represent the reactor number (indicating the biological replicate) and the sampling time point (denoting the growth phase): ME (mid-exponential), LE (late-exponential), ES (early-stationary) and MS (mid-stationary). Overall, the biological replicates from ES and MS cluster well as well as ME and LE clustered well, which is expected given the minimal proteome differences between these growth phases. A more pronounced change was only observed between exponential and stationary growth phases.

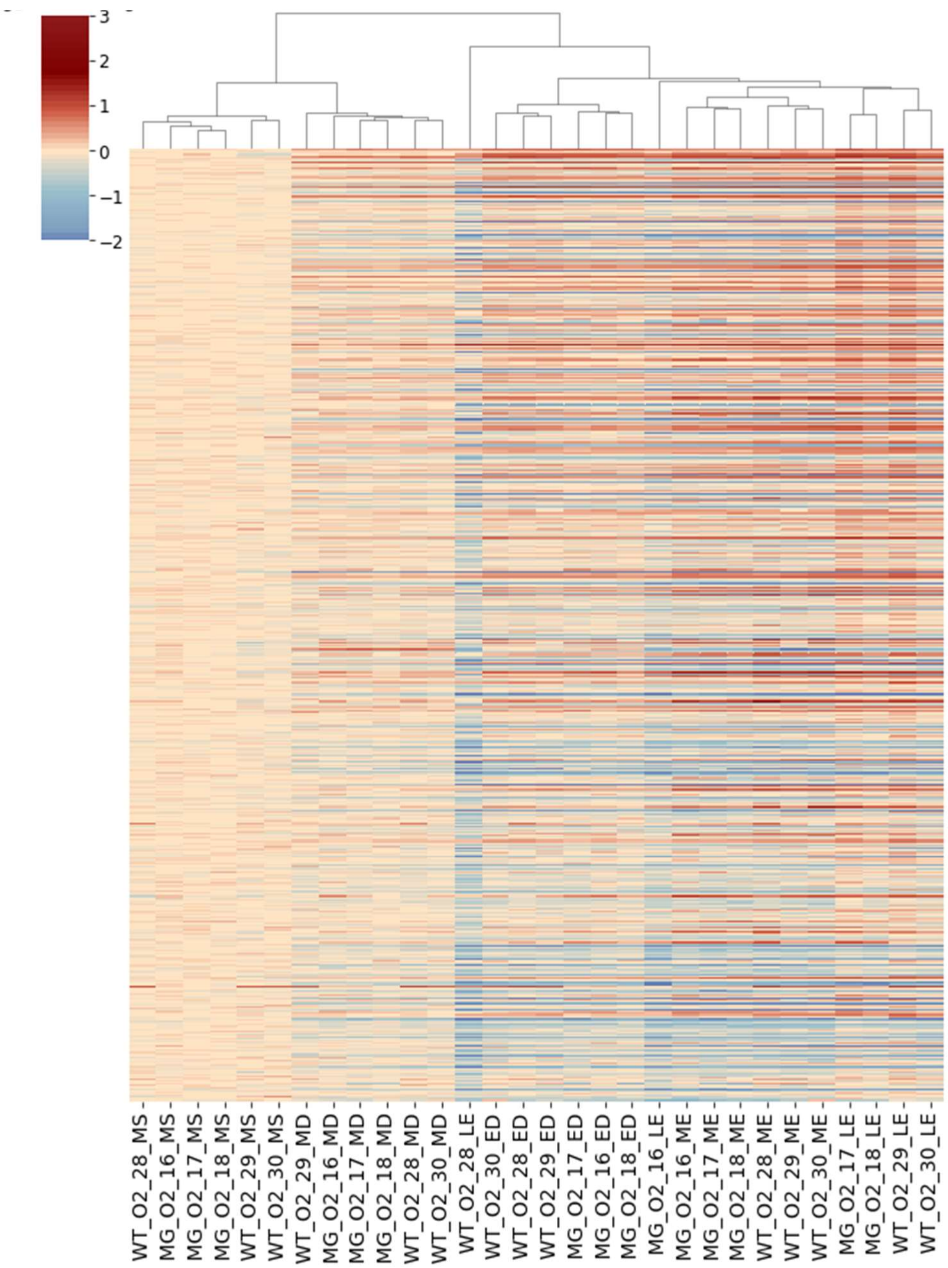

SI Figure 3.5. **Cluster analysis of replicates and growth phase time points from aerobically (O2) grown wild-type (WT) and minimal glycolysis (MG) strain.** Cluster analysis was conducted using the average linkage method and Euclidean distances. The log2 of the ratio to the bridging sample was calculated and visualized such that a ratio of 1 is colored white, a negative ratio is colored blue, and a positive ratio is colored red. The labels of the columns represent the reactor number (which indicates the biological replicate) and the sampling time point (which represents the growth phase): ME (mid-exponential), LE (late-exponential), ED (early-diauxic), MD (mid-diauxic shift), ES (early-stationary), and MS (mid-stationary). The biological replicates of the individual growth phase for both strains (WT and MG) show a clear grouping, except for some variations of the LE time point replicates. The clustering moreover confirms the neglectable proteome differences between the WT and the MG mutant strain.

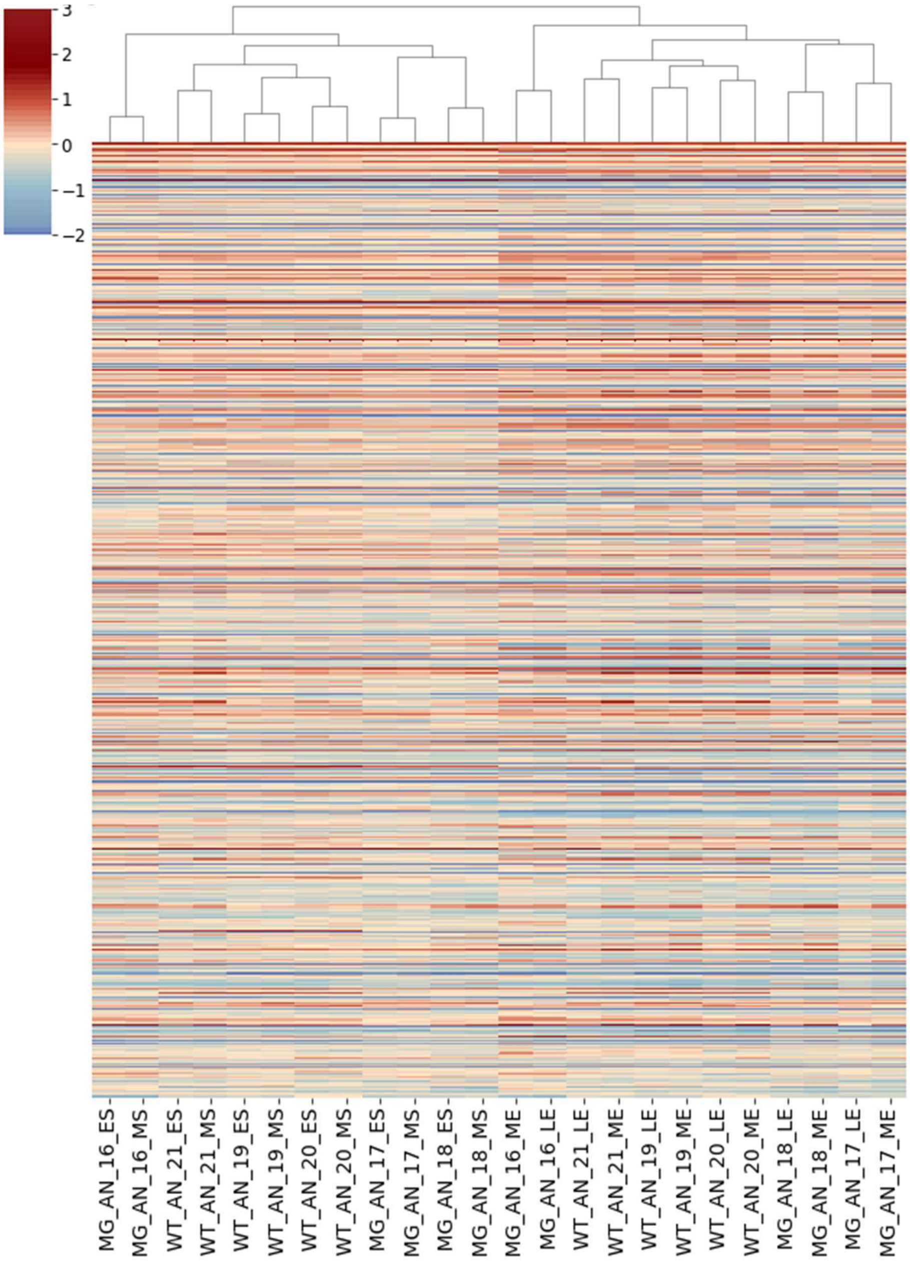

SI Figure 3.6. **Cluster analysis of replicates and growth phase time points from anaerobically (AN) grown wild-type (WT) and minimal glycolysis (MG) strain.** Cluster analysis was conducted using the average linkage method and Euclidean distances. The log2 of the ratio to the bridging sample was calculated and visualized such that a ratio of 1 is colored white, a negative ratio is colored blue, and a positive ratio is colored red. The column labels represent the strain (WT or MG), the reactor number (indicating the biological replicate) and the sampling time point (denoting the growth phase): ME (mid-exponential), LE (late-exponential), ES (early-stationary) and MS (mid-stationary). Overall, the biological replicates from ES and MS as well as ME and LE of both strains grouped together, which is expected given the minimal proteome differences between these growth phases, and strains respectively. The proteome differences between exponential and stationary growth phases were found to be significantly more pronounced.

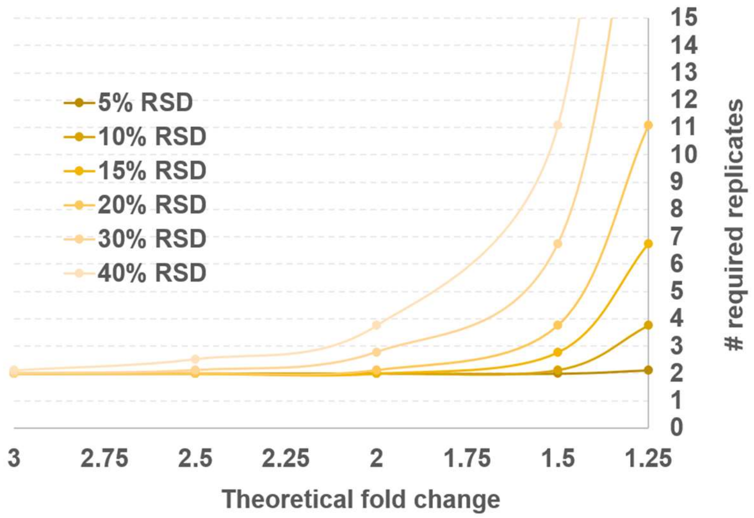

SI Figure 4. Line graphs showing the required number of replicates for different experimental variations (5, 10, 15, 20, 30 and 40%) as outlined in SI Table 2. The line graph visualises the required number of biological replicates for different experimental variations (shown as individual lines) that allow to determine different fold change levels (with a power of 0.8, and alpha=0.05). For lower RSD values (5, 10 and 15%) a 1.5 fold change, and for larger RSD values (20 and 30%) at least 3 biological replicates are required.

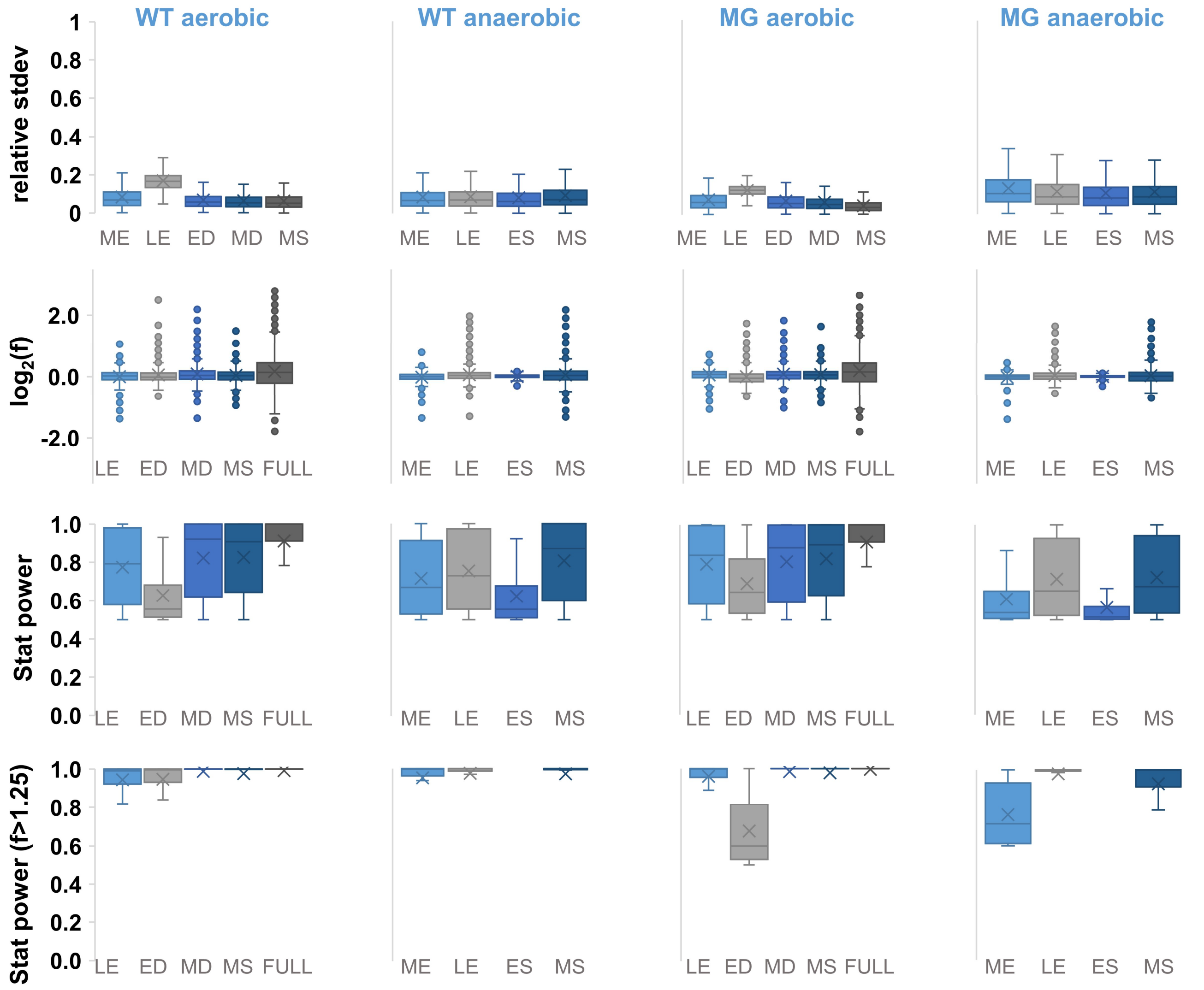

SI Figure 5. Experimental variation and statistical power for performed TMT experiments. The boxplots demonstrate that the overall experimental variation was very low (relative standard deviation mostly in range of 5–15%), and that the statistical power for the experiments (when considering proteins with a fold change >1.25 was generally very high (except for 2 conditions, median>0.9). The graphs consider proteins that were consistently detected across the growth experiment and all 3 biological replicates. A) The boxplots (top) show the relative standard deviation (RSD) for the biological replicates of the individual conditions, from left to right: WT aerobic, WT anaerobic, MG aerobic and MG anaerobic (individual conditions aerobic: ME=mid-exponential, LE=late exponential, ED=early diauxic, MD=mid-diauxic, MS=mid-stationary; individual conditions anaerobic: ME=mid-exponential, LE=late exponential, ES=early stationary, MS=mid stationary). The relative standard deviations (RSDs) for individual conditions are generally low (mostly in the range of 5–15%). Only for the aerobic LE growth time point the RSD was slightly elevated (approx. 20%). B) The boxplots in the second row (from the top) show the $\log_2$(fold change) of the individual growth stage transitions. From left to right the boxplots show: WT aerobic, WT anaerobic, MG aerobic and MG anaerobic (aerobic transitions: LE=ME→LE, ED=LE→ED, MD=ED→MD, MS=MD→MS, FULL=ME→MS; anaerobic transitions: LE=ME→LE, ES=LE→ES, MS=MD→MS, FULL=ME→MS). C) The boxplots in third row (from the top) show the statistical power obtained for the fold change values and RSDs of the individual proteins of the respective growth transitions, for n=3 and alpha=0.05. From left to right, the boxplots show: WT aerobic, WT anaerobic, MG aerobic and MG anaerobic (aerobic transitions: LE=ME→LE, ED=LE→ED, MD=ED→MD, MS=MD→MS, FULL=ME→MS; anaerobic transitions: LE=ME→LE, ES=LE→ES, MS=MD→MS, FULL=ME→MS). D) The boxplots in the bottom row show the statistical power calculated for the fold change and RSD obtained for proteins with a fold change >1.25, using n=3 and alpha=0.05 (from left to right: WT aerobic, WT anaerobic, MG aerobic and MG anaerobic; aerobic transitions: LE=ME→LE, ED=LE→ED, MD=ED→MD, MS=MD→MS, FULL=ME→MS; anaerobic transitions: LE=ME→LE, ES=LE→ES, MS=MD→MS, FULL=ME→MS). The power calculations were performed using the python "statsmodels module" (https://www.statsmodels.org, stats.power.TTestIndPower.solve_power().

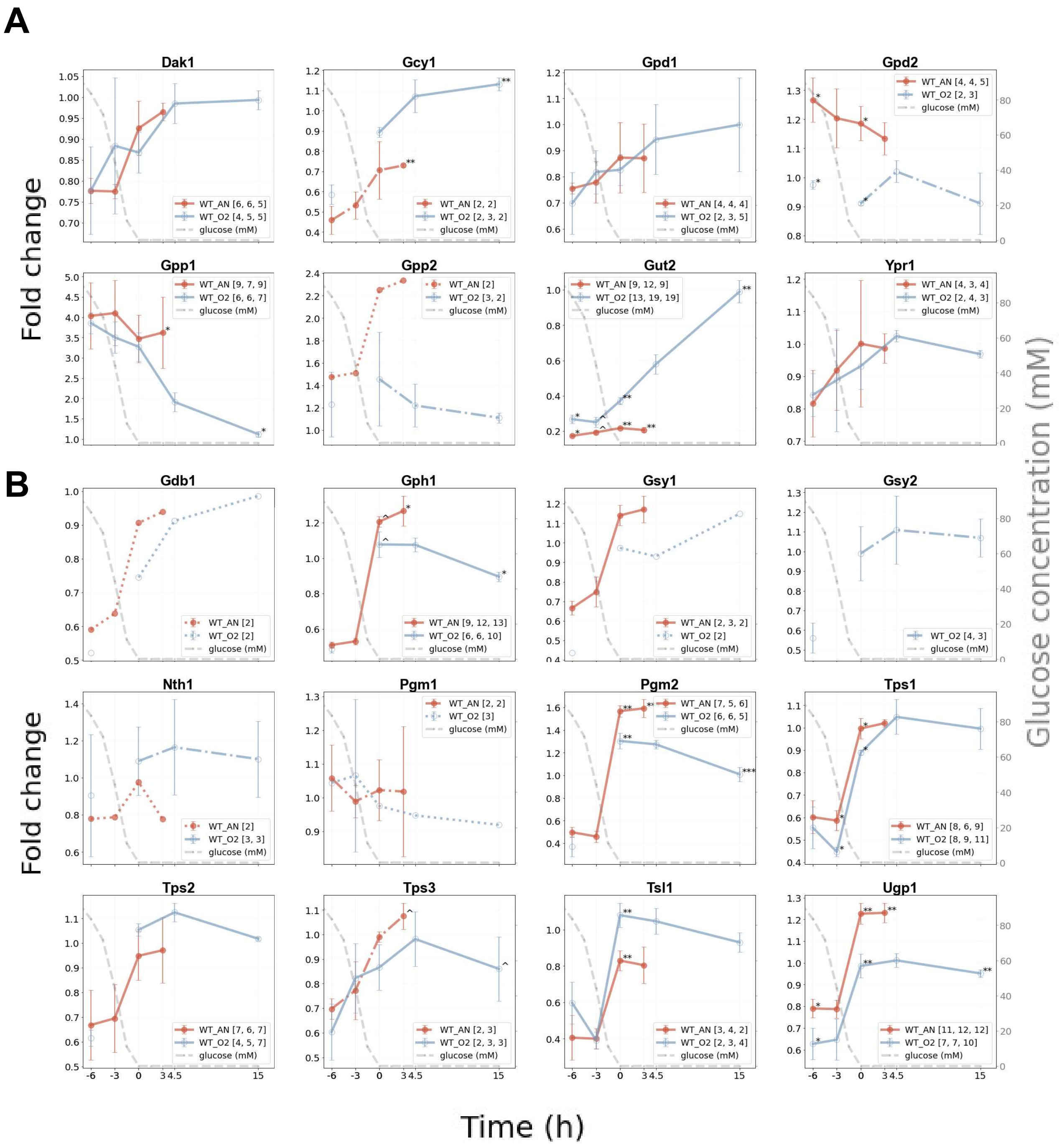

SI Figure 6. Protein fold change line graphs for the glycerol, glycogen and trehalose metabolism during aerobic and anaerobic growth for the control yeast CEN.PK113-7D. The biological-replicate-averaged fold-change values were plotted against the time relative to glucose depletion in hours for proteins involved in glycerol (A) and glycogen and trehalose (B) metabolism. The different colours of the line graphs represent: "orange" the control strain under anaerobic conditions (WT_AN) and "light blue" the control strain under aerobic conditions (WT_O2). The error bars show the standard deviation of the mean of the three biological replicates. The grey dashed line represents the glucose concentration over time (mM, secondary y-axis). The number of quantified peptides per biological replicate are indicated in brackets. Asterisks (*) and circumflexes (ˆ) indicate the significance between the aerobic and anaerobic experiments, which are as follows: $p < 0.001$ (***), $p < 0.01$ (**), $p < 0.05$ (*), and $p < 0.1$ (ˆ).

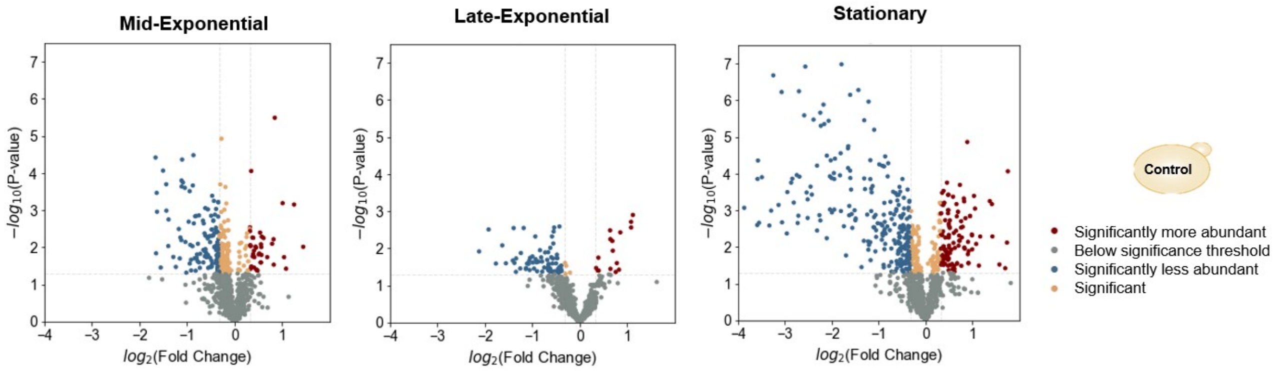

SI Figure 7. Volcano plots showing the global proteome changes between aerobically and anaerobically cultured control yeast CEN.PK113-7D. The log2 of the abundance fold change between the two conditions (normalised to the aerobic experiments) was plotted against the -log10 of the p-value. The mid-exponential (ME), late-exponential (LE) and mid-stationary (MS) phases were compared. P-value threshold <0.05, fold change threshold >1.5 (log2 fold change threshold +/- 0.32).

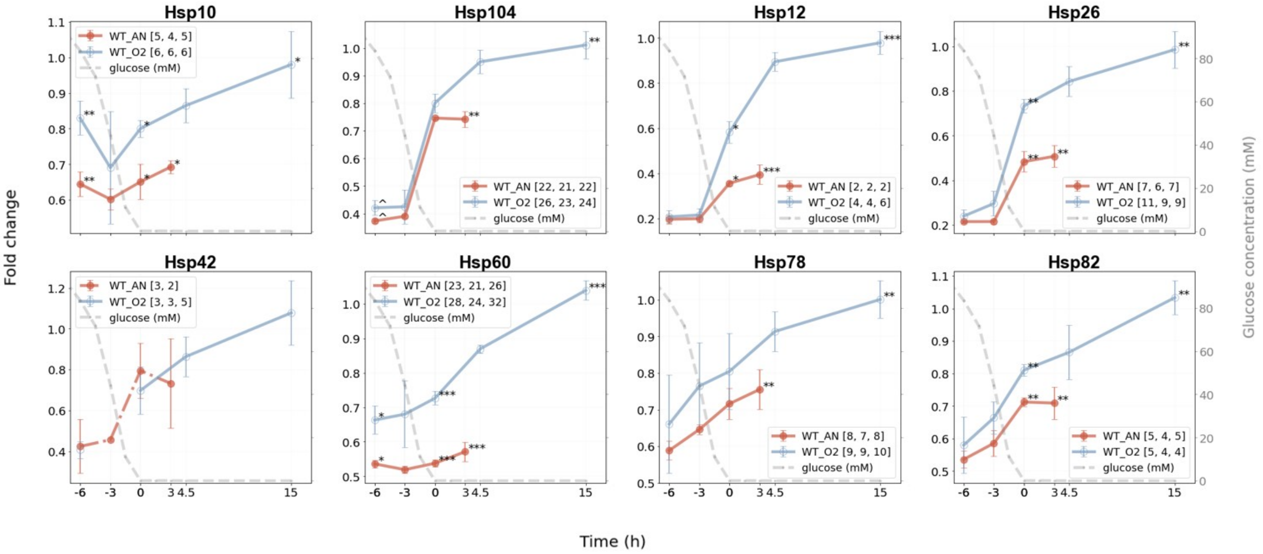

SI Figure 8. Fold changes line graphs for heat shock proteins observed for the control yeast CEN.PK113-7D under aerobic and anaerobic conditions. The biological-replicate-averaged fold changes were plotted against the time relative to glucose depletion (0 h) in hours. Orange: CEN.PK113-7D under anaerobic conditions, light blue CEN.PK113-7D under aerobic conditions. The error bars show the standard deviation of the mean of the three biological replicates. In the legend, the numbers between brackets represent the number of unique peptides that were found in each biological replicate. The grey dashed line represents the glucose concentration over time (mM, secondary y-axis). Asterisks (*) and circumflexes (ˆ) indicate the significance between the aerobic and anaerobic experiments, which are: $p < 0.001$ (***), $p < 0.01$ (**), $p < 0.05$ (*), and $p < 0.1$ (ˆ).

ATP synthesis

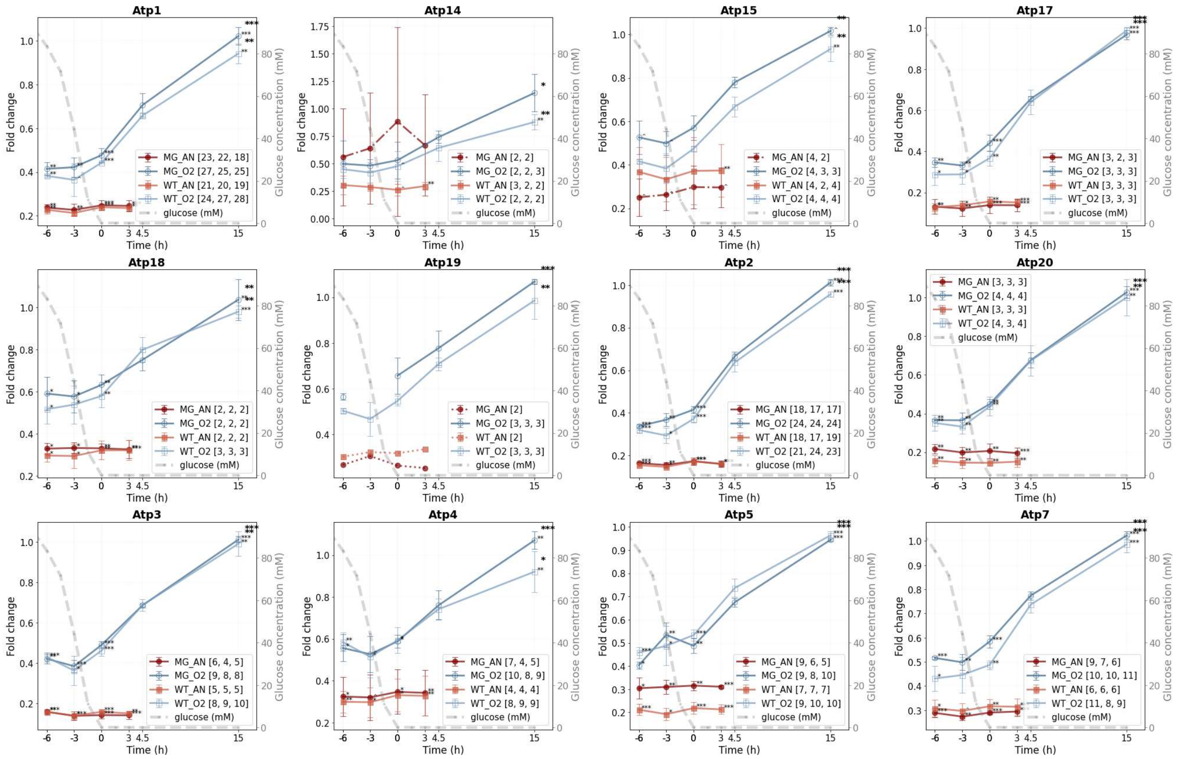

SI Figure 9.1. Fold change line graphs for proteins of selected pathways for the control yeast and the MG strain under aerobic and anaerobic conditions. The biological-replicate-averaged FCs of CEN.PK113-7D and IMX372 (MG) were plotted against the time relative to glucose depletion (0 h) in hours. Red: the MG strain under anaerobic conditions, dark blue: the MG strain under aerobic conditions, orange: the control strain under anaerobic conditions, light blue: the control strain under aerobic conditions. The error bars show the standard deviation of the mean of the three biological replicates. In the legend, the numbers between brackets represent the number of unique peptides that were found in each biological replicate. The grey dashed line represents the glucose concentration over time (mM, secondary y-axis). Asterisks (*) and circumflexes (^) indicate the significance between either the (an)aerobic experiments (black annotation) or between the ME and MS phase. P-value levels are: $p < 0.001$ (***), $p < 0.01$ (**), $p < 0.05$ (*), and $p < 0.1$ (^).

Respiration

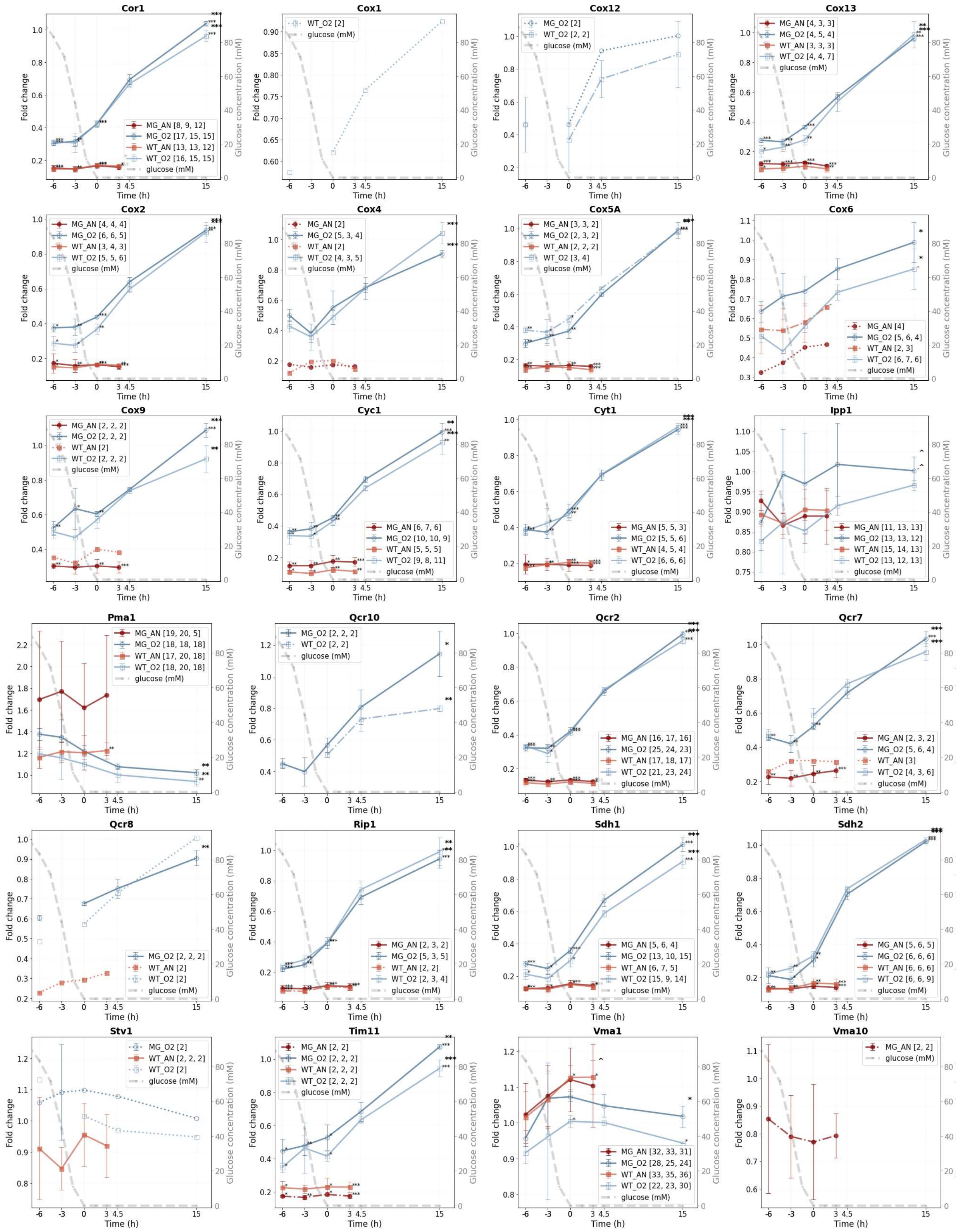

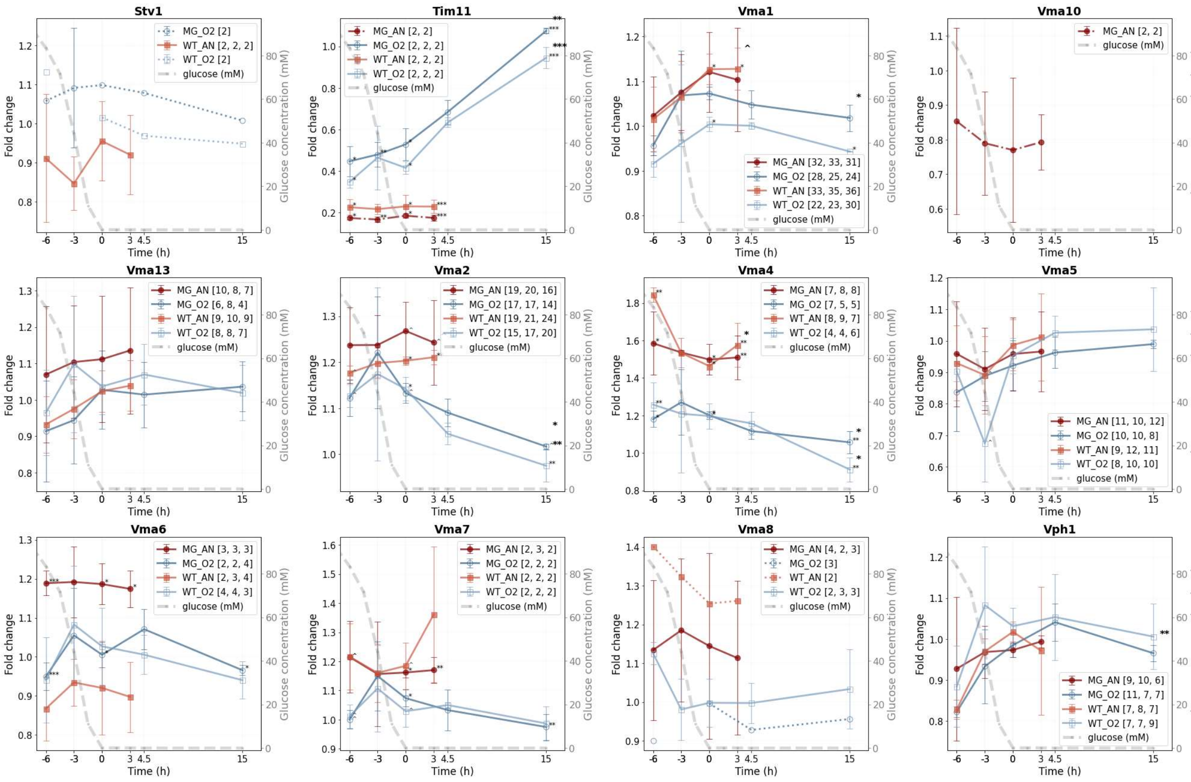

SI Figure 9.2. Fold change line graphs for proteins of selected pathways for the control yeast and the MG strain under aerobic and anaerobic conditions. The biological-replicate-averaged FCs of CEN.PK113-7D and IMX372 (MG) were plotted against the time relative to glucose depletion (0 h) in hours. Red: the MG strain under anaerobic conditions, dark blue: the MG strain under aerobic conditions, orange: the control strain under anaerobic conditions, light blue: the control strain under aerobic conditions. The error bars show the standard deviation of the mean of the three biological replicates. In the legend, the numbers between brackets represent the number of unique peptides that were found in each biological replicate. The grey dashed line represents the glucose concentration over time (mM, secondary y-axis). Asterisks (*) and circumflexes (^) indicate the significance between either the (an)aerobic experiments (black annotation) or between the ME and MS phase. P-value levels are: $p < 0.001$ (***), $p < 0.01$ (**), $p < 0.05$ (*), and $p < 0.1$ (^).

Plasma membrane transport

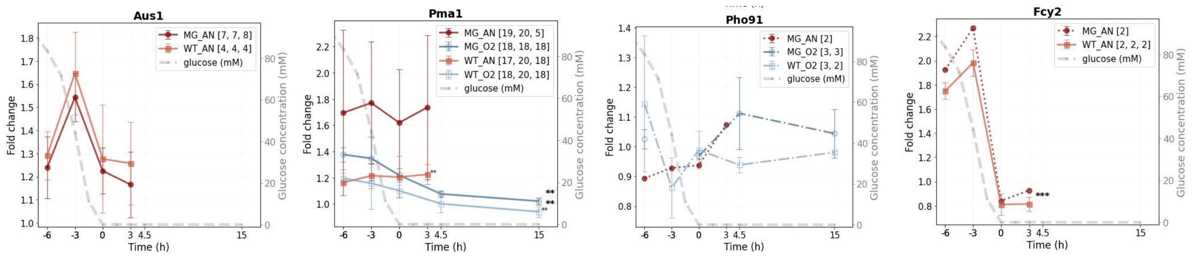

SI Figure 9.3. Fold change line graphs for proteins of selected pathways for the control yeast and the MG strain under aerobic and anaerobic conditions. The biological-replicate-averaged FCs of CEN.PK113-7D and IMX372 (MG) were plotted against the time relative to glucose depletion (0 h) in hours. Red: the MG strain under anaerobic conditions, dark blue: the MG strain under aerobic conditions, orange: the control strain under anaerobic conditions, light blue: the control strain under aerobic conditions. The error bars show the standard deviation of the mean of the three biological replicates. In the legend, the numbers between brackets represent the number of unique peptides that were found in each biological replicate. The grey dashed line represents the glucose concentration over time (mM, secondary y-axis). Asterisks (*) and circumflexes (^) indicate the significance between either the (an)aerobic experiments (black annotation) or between the ME and MS phase. P-value levels are: $p < 0.001$ (***), $p < 0.01$ (**), $p < 0.05$ (*), and $p < 0.1$ (^).

Fatty acid metabolism

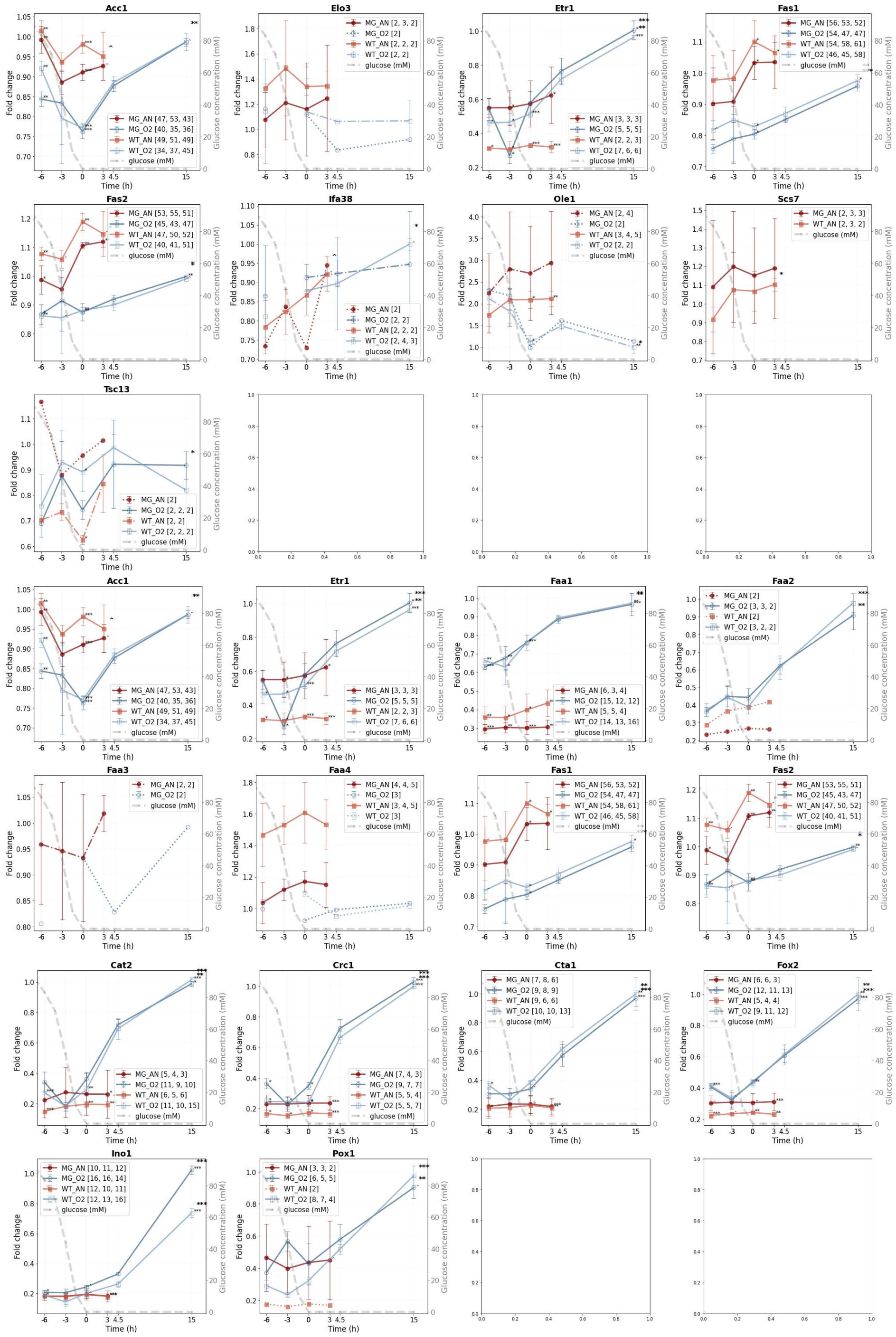

SI Figure 9.4. Fold change line graphs for proteins of selected pathways for the control yeast and the MG strain under aerobic and anaerobic conditions. The biological-replicate-averaged FCs of CEN.PK113-7D and IMX372 (MG) were plotted against the time relative to glucose depletion (0 h) in hours. Red: the MG strain under anaerobic conditions, dark blue: the MG strain under aerobic conditions, orange: the control strain under anaerobic conditions, light blue: the control strain under aerobic conditions. The error bars show the standard deviation of the mean of the three biological replicates. In the legend, the numbers between brackets represent the number of unique peptides that were found in each biological replicate. The grey dashed line represents the glucose concentration over time (mM, secondary y-axis). Asterisks (*) and circumflexes (^) indicate the significance between either the (an)aerobic experiments (black annotation) or between the ME and MS phase. P-value levels are: p < 0.001 (***), p < 0.01 (**), p < 0.05 (*), and p < 0.1 (^).

Autophagy

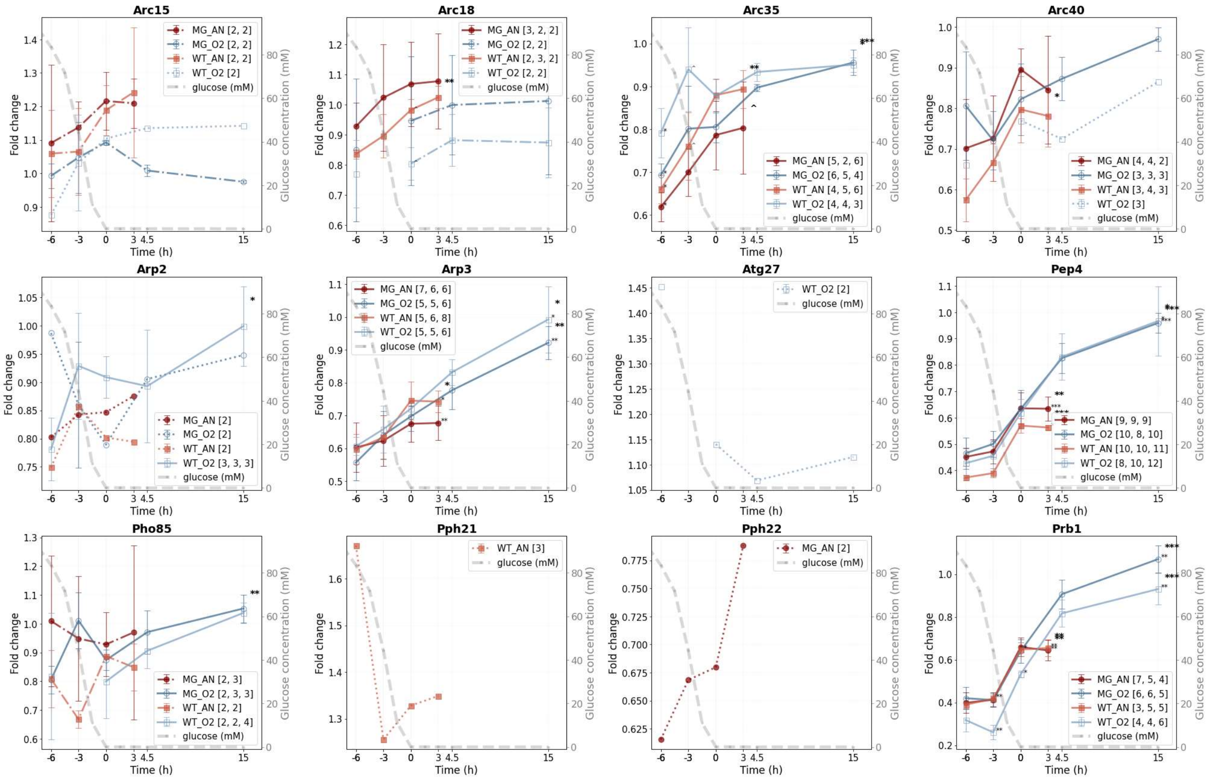
Arc15
MG_AN [2, 2]
MG_O2 [2, 2]
WT_AN [2, 2]
WT_O2 [2]
glucose (mM)
Arc18
MG_AN [3, 2, 2]
MG_O2 [2, 2]
WT_AN [2, 3, 2]
WT_O2 [2, 2]
glucose (mM)
Arc35
MG_AN [5, 2, 6]
MG_O2 [6, 5, 4]
WT_AN [4, 5, 6]
WT_O2 [4, 4, 3]
glucose (mM)
Arc40
MG_AN [4, 4, 2]
MG_O2 [3, 3, 3]
WT_AN [3, 4, 3]
WT_O2 [3]
glucose (mM)
Arp2
MG_AN [2]
MG_O2 [2]
WT_AN [2]
WT_O2 [3, 3, 3]
glucose (mM)
Arp3
MG_AN [7, 6, 6]
MG_O2 [5, 5, 6]
WT_AN [5, 6, 8]
WT_O2 [5, 5, 6]
glucose (mM)
Atg27
WT_O2 [2]
glucose (mM)
Pep4
MG_AN [9, 9, 9]
MG_O2 [10, 8, 10]
WT_AN [10, 10, 11]
WT_O2 [8, 10, 12]
glucose (mM)
Pho85
MG_AN [2, 3]
MG_O2 [2, 3, 3]
WT_AN [2, 2]
WT_O2 [2, 2, 4]
glucose (mM)
Pph21
WT_AN [3]
glucose (mM)
Pph22
MG_AN [2]
glucose (mM)
Prb1
MG_AN [7, 5, 4]
MG_O2 [6, 6, 5]
WT_AN [3, 5, 5]
WT_O2 [4, 4, 6]
glucose (mM)
Fold change
Glucose concentration (mM)
Time (h)

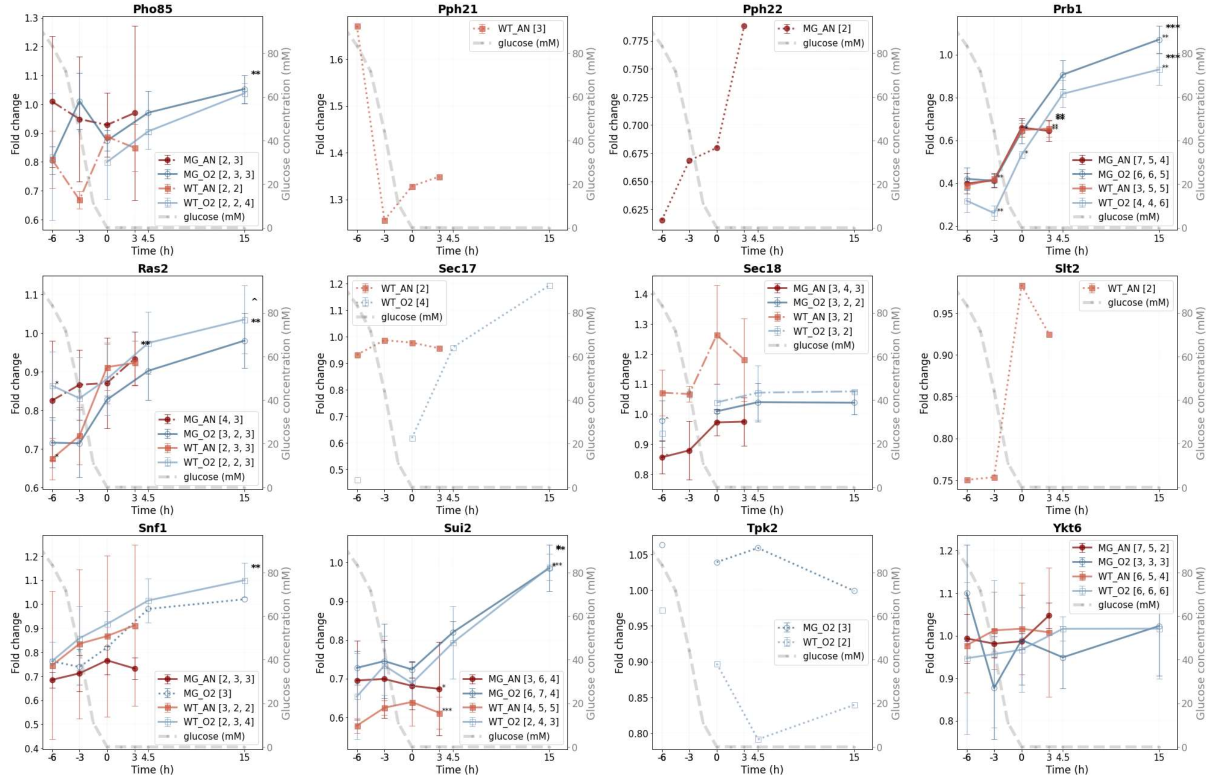
Pho85
MG_AN [2, 3]
MG_O2 [2, 3, 3]
WT_AN [2, 2]
WT_O2 [2, 2, 4]
glucose (mM)
Pph21
WT_AN [3]
glucose (mM)
Pph22
MG_AN [2]
glucose (mM)
Prb1
MG_AN [7, 5, 4]
MG_O2 [6, 6, 5]
WT_AN [3, 5, 5]
WT_O2 [4, 4, 6]
glucose (mM)
Ras2
MG_AN [4, 3]
MG_O2 [3, 2, 3]
WT_AN [2, 3, 3]
WT_O2 [2, 2, 3]
glucose (mM)
Sec17
WT_AN [2]
WT_O2 [4]
glucose (mM)
Sec18
MG_AN [3, 4, 3]
MG_O2 [3, 2, 2]
WT_AN [3, 2]
WT_O2 [3, 2]
glucose (mM)
Slt2
WT_AN [2]
glucose (mM)
Snf1
MG_AN [2, 3, 3]
MG_O2 [3]
WT_AN [3, 2, 2]
WT_O2 [2, 3, 4]
glucose (mM)
Sui2
MG_AN [3, 6, 4]
MG_O2 [6, 7, 4]
WT_AN [4, 5, 5]
WT_O2 [2, 4, 3]
glucose (mM)
Tpk2
MG_O2 [3]
WT_O2 [2]
glucose (mM)
Ykt6
MG_AN [7, 5, 2]
MG_O2 [3, 3, 3]
WT_AN [6, 5, 4]
WT_O2 [6, 6, 6]
glucose (mM)
Fold change
Glucose concentration (mM)
Time (h)

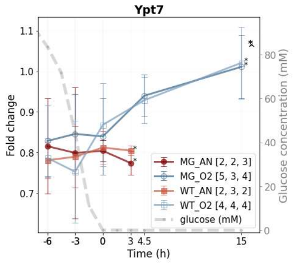

SI Figure 9.5. Fold change line graphs for proteins of selected pathways for the control yeast and the MG strain under aerobic and anaerobic conditions. The biological-replicate-averaged FCs of CEN.PK113-7D and IMX372 (MG) were plotted against the time relative to glucose depletion (0 h) in hours. Red: the MG strain under anaerobic conditions, dark blue: the MG strain under aerobic conditions, orange: the control strain under anaerobic conditions, light blue: the control strain under aerobic conditions. The error bars show the standard deviation of the mean of the three biological replicates. In the legend, the numbers between brackets represent the number of unique peptides that were found in each biological replicate. The grey dashed line represents the glucose concentration over time (mM, secondary y-axis). Asterisks (*) and circumflexes (ˆ) indicate the significance between either the (an)aerobic experiments (black annotation) or between the ME and MS phase. P-value levels are: $p < 0.001$ (***), $p < 0.01$ (**), $p < 0.05$ (*), and $p < 0.1$ (ˆ).

Ribosomes

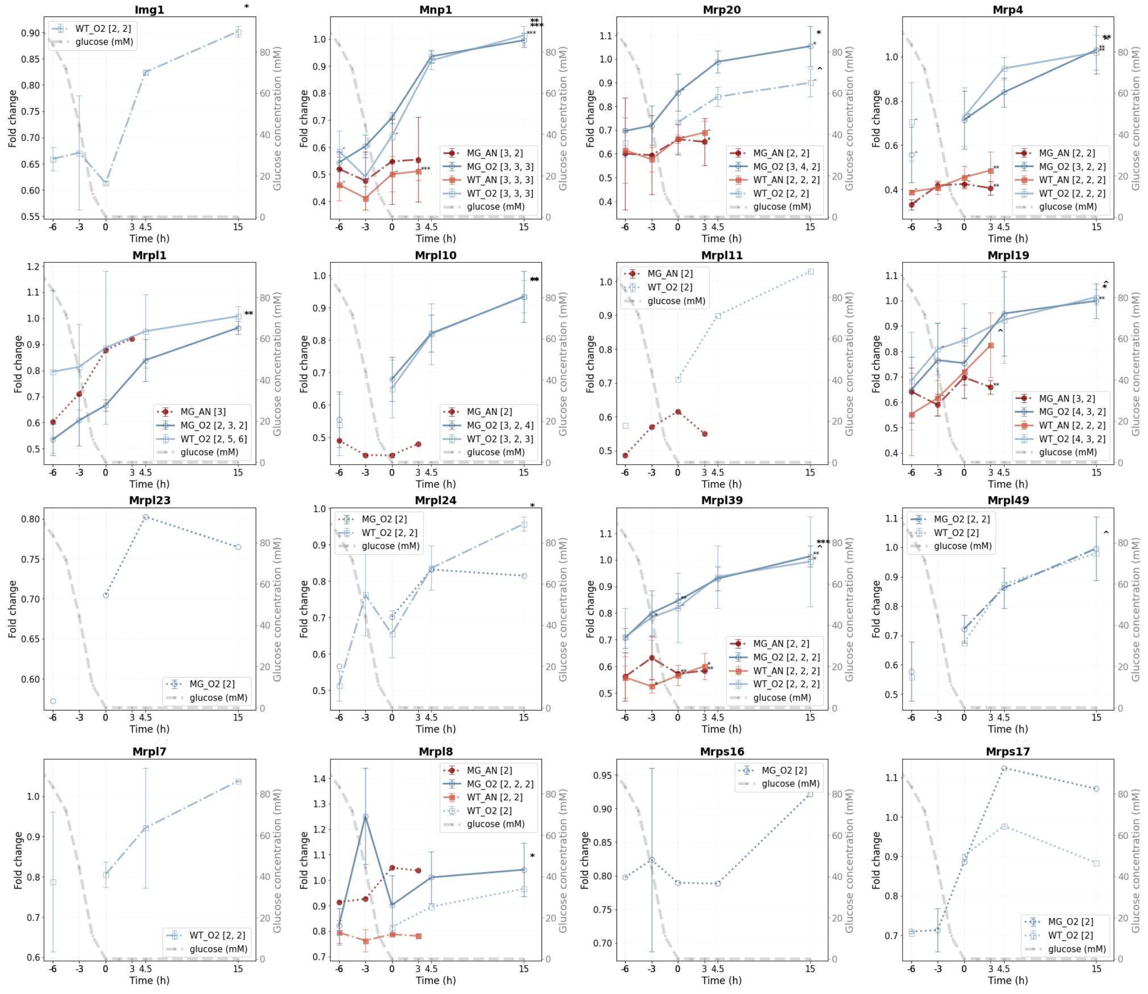

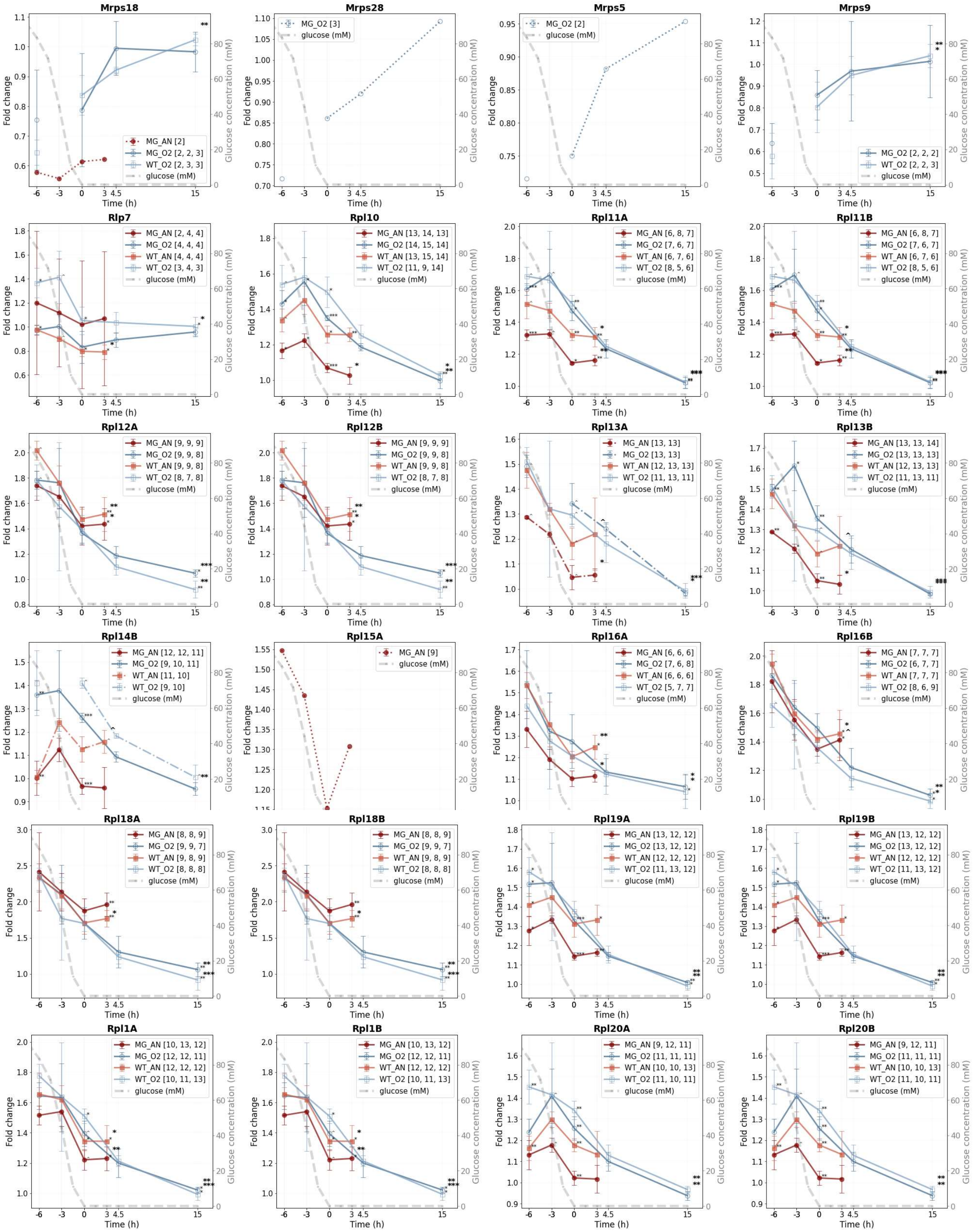
Mrps18
MG_AN [2]
MG_O2 [2, 2, 3]
WT_O2 [2, 3, 3]
glucose (mM)
Mrps28
MG_O2 [3]
glucose (mM)
Mrps5
MG_O2 [2]
glucose (mM)
Mrps9
MG_O2 [2, 2, 2]
WT_O2 [2, 2, 3]
glucose (mM)
Rlp7
MG_AN [2, 4, 4]
MG_O2 [4, 4, 4]
WT_AN [4, 4, 4]
WT_O2 [3, 4, 3]
glucose (mM)
Rpl10
MG_AN [13, 14, 13]
MG_O2 [14, 15, 14]
WT_AN [13, 15, 14]
WT_O2 [11, 9, 14]
glucose (mM)
Rpl11A
MG_AN [6, 8, 7]
MG_O2 [7, 6, 7]
WT_AN [6, 7, 6]
WT_O2 [8, 5, 6]
glucose (mM)
Rpl11B
MG_AN [6, 8, 7]
MG_O2 [7, 6, 7]
WT_AN [6, 7, 6]
WT_O2 [8, 5, 6]
glucose (mM)
Rpl12A
MG_AN [9, 9, 9]
MG_O2 [9, 9, 8]
WT_AN [9, 9, 8]
WT_O2 [8, 7, 8]
glucose (mM)
Rpl12B
MG_AN [9, 9, 9]
MG_O2 [9, 9, 8]
WT_AN [9, 9, 8]
WT_O2 [8, 7, 8]
glucose (mM)
Rpl13A
MG_AN [13, 13]
MG_O2 [13, 13]
WT_AN [12, 13, 13]
WT_O2 [11, 13, 11]
glucose (mM)
Rpl13B
MG_AN [13, 13, 14]
MG_O2 [13, 13, 13]
WT_AN [12, 13, 13]
WT_O2 [11, 13, 11]
glucose (mM)
Rpl14B
MG_AN [12, 12, 11]
MG_O2 [9, 10, 11]
WT_AN [11, 10]
WT_O2 [9, 10]
glucose (mM)
Rpl15A
MG_AN [9]
glucose (mM)
Rpl16A
MG_AN [6, 6, 6]
MG_O2 [7, 6, 8]
WT_AN [6, 6, 6]
WT_O2 [5, 7, 7]
glucose (mM)
Rpl16B
MG_AN [7, 7, 7]
MG_O2 [6, 7, 7]
WT_AN [7, 7, 7]
WT_O2 [8, 6, 9]
glucose (mM)
Rpl18A
MG_AN [8, 8, 9]
MG_O2 [9, 9, 7]
WT_AN [9, 8, 9]
WT_O2 [8, 8, 8]
glucose (mM)
Rpl18B
MG_AN [8, 8, 9]
MG_O2 [9, 9, 7]
WT_AN [9, 8, 9]
WT_O2 [8, 8, 8]
glucose (mM)
Rpl19A
MG_AN [13, 12, 12]
MG_O2 [13, 12, 12]
WT_AN [12, 12, 12]
WT_O2 [11, 13, 12]
glucose (mM)
Rpl19B
MG_AN [13, 12, 12]
MG_O2 [13, 12, 12]
WT_AN [12, 12, 12]
WT_O2 [11, 13, 12]
glucose (mM)
Rpl1A
MG_AN [10, 13, 12]
MG_O2 [12, 12, 11]
WT_AN [12, 12, 12]
WT_O2 [10, 11, 13]
glucose (mM)
Rpl1B
MG_AN [10, 13, 12]
MG_O2 [12, 12, 11]
WT_AN [12, 12, 12]
WT_O2 [10, 11, 13]
glucose (mM)
Rpl20A
MG_AN [9, 12, 11]
MG_O2 [11, 11, 11]
WT_AN [10, 10, 13]
WT_O2 [11, 10, 11]
glucose (mM)
Rpl20B
MG_AN [9, 12, 11]
MG_O2 [11, 11, 11]
WT_AN [10, 10, 13]
WT_O2 [11, 10, 11]
glucose (mM)
Fold change
Time (h)
Glucose concentration (mM)

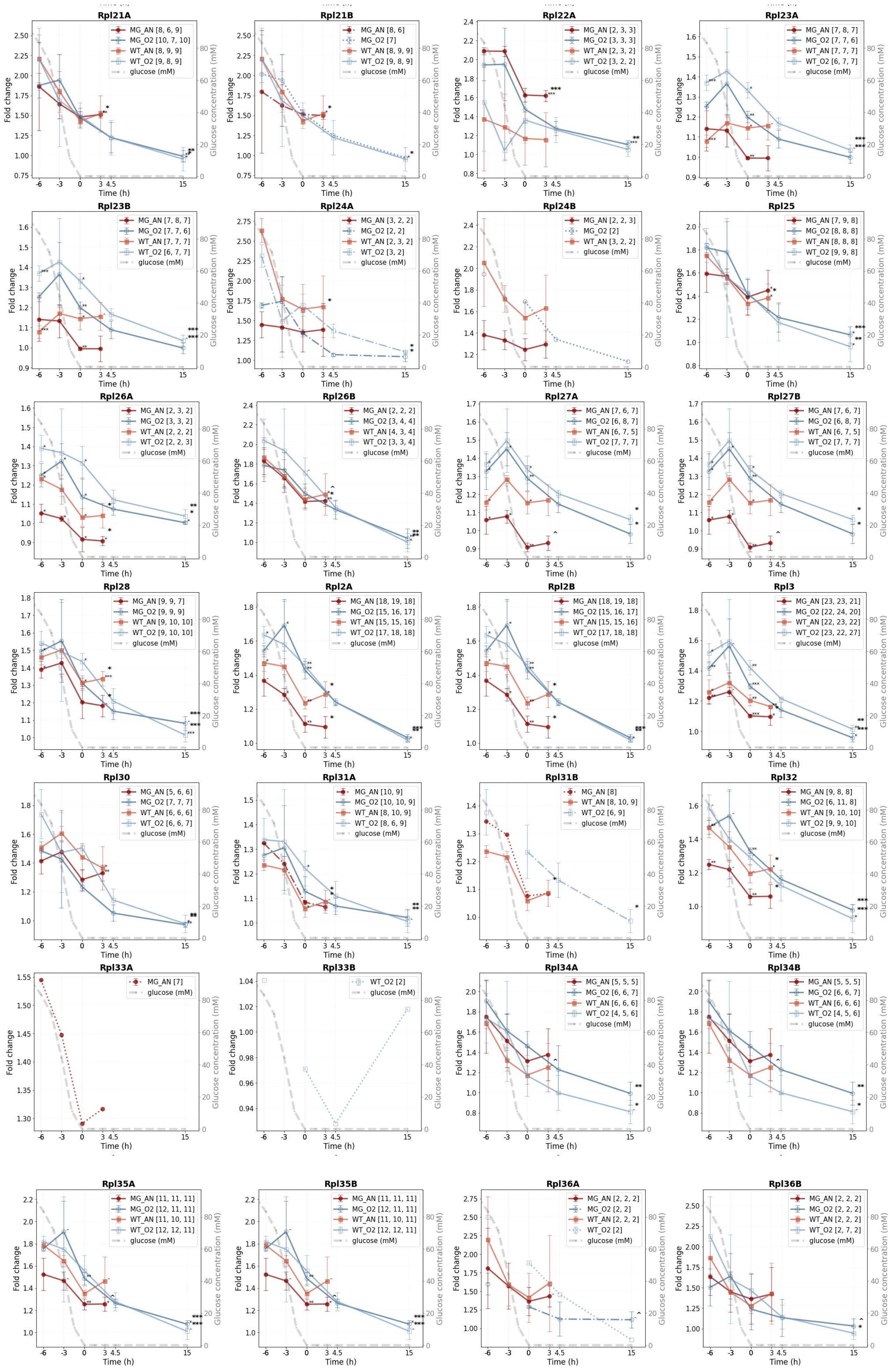
Rpl21A
MG_AN [8, 6, 9]
MG_O2 [10, 7, 10]
WT_AN [8, 9, 9]
WT_O2 [9, 8, 9]
glucose (mM)
Rpl21B
MG_AN [8, 6]
MG_O2 [7]
WT_AN [8, 9, 9]
WT_O2 [9, 8, 9]
glucose (mM)
Rpl22A
MG_AN [2, 3, 3]
MG_O2 [3, 3, 3]
WT_AN [2, 3, 2]
WT_O2 [3, 2, 2]
glucose (mM)
Rpl23A
MG_AN [7, 8, 7]
MG_O2 [7, 7, 6]
WT_AN [7, 7, 7]
WT_O2 [6, 7, 7]
glucose (mM)
Rpl23B
MG_AN [7, 8, 7]
MG_O2 [7, 7, 6]
WT_AN [7, 7, 7]
WT_O2 [6, 7, 7]
glucose (mM)
Rpl24A
MG_AN [3, 2, 2]
MG_O2 [2, 2]
WT_AN [2, 3, 2]
WT_O2 [3, 2]
glucose (mM)
Rpl24B
MG_AN [2, 2, 3]
MG_O2 [2]
WT_AN [3, 2, 2]
glucose (mM)
Rpl25
MG_AN [7, 9, 8]
MG_O2 [8, 8, 8]
WT_AN [8, 8, 8]
WT_O2 [9, 9, 8]
glucose (mM)
Rpl26A
MG_AN [2, 3, 2]
MG_O2 [3, 3, 2]
WT_AN [2, 2, 2]
WT_O2 [2, 2, 3]
glucose (mM)
Rpl26B
MG_AN [2, 2, 2]
MG_O2 [3, 4, 4]
WT_AN [4, 3, 4]
WT_O2 [3, 3, 4]
glucose (mM)
Rpl27A
MG_AN [7, 6, 7]
MG_O2 [6, 8, 7]
WT_AN [6, 7, 5]
WT_O2 [7, 7, 7]
glucose (mM)
Rpl27B
MG_AN [7, 6, 7]
MG_O2 [6, 8, 7]
WT_AN [6, 7, 5]
WT_O2 [7, 7, 7]
glucose (mM)
Rpl28
MG_AN [9, 9, 7]
MG_O2 [9, 9, 9]
WT_AN [9, 10, 10]
WT_O2 [9, 10, 10]
glucose (mM)
Rpl2A
MG_AN [18, 19, 18]
MG_O2 [15, 16, 17]
WT_AN [15, 15, 16]
WT_O2 [17, 18, 18]
glucose (mM)
Rpl2B
MG_AN [18, 19, 18]
MG_O2 [15, 16, 17]
WT_AN [15, 15, 16]
WT_O2 [17, 18, 18]
glucose (mM)
Rpl3
MG_AN [23, 23, 21]
MG_O2 [22, 24, 20]
WT_AN [22, 23, 22]
WT_O2 [23, 22, 27]
glucose (mM)
Rpl30
MG_AN [5, 6, 6]
MG_O2 [7, 7, 7]
WT_AN [6, 6, 6]
WT_O2 [6, 6, 7]
glucose (mM)
Rpl31A
MG_AN [10, 9]
MG_O2 [10, 10, 9]
WT_AN [8, 10, 9]
WT_O2 [8, 6, 9]
glucose (mM)
Rpl31B
MG_AN [8]
WT_AN [8, 10, 9]
WT_O2 [6, 9]
glucose (mM)
Rpl32
MG_AN [9, 8, 8]
MG_O2 [6, 11, 8]
WT_AN [9, 10, 10]
WT_O2 [9, 9, 10]
glucose (mM)
Rpl33A
MG_AN [7]
glucose (mM)
Rpl33B
WT_O2 [2]
glucose (mM)
Rpl34A
MG_AN [5, 5, 5]
MG_O2 [6, 6, 7]
WT_AN [6, 6, 6]
WT_O2 [4, 5, 6]
glucose (mM)
Rpl34B
MG_AN [5, 5, 5]
MG_O2 [6, 6, 7]
WT_AN [6, 6, 6]
WT_O2 [4, 5, 6]
glucose (mM)
Rpl35A
MG_AN [11, 11, 11]
MG_O2 [12, 11, 11]
WT_AN [11, 10, 11]
WT_O2 [12, 12, 11]
glucose (mM)
Rpl35B
MG_AN [11, 11, 11]
MG_O2 [12, 11, 11]
WT_AN [11, 10, 11]
WT_O2 [12, 12, 11]
glucose (mM)
Rpl36A
MG_AN [2, 2, 2]
MG_O2 [2, 2]
WT_AN [2, 2, 2]
WT_O2 [2]
glucose (mM)
Rpl36B
MG_AN [2, 2, 2]
MG_O2 [2, 2, 2]
WT_AN [2, 2, 2]
WT_O2 [2, 7, 2]
glucose (mM)
Fold change
Glucose concentration (mM)
Time (h)

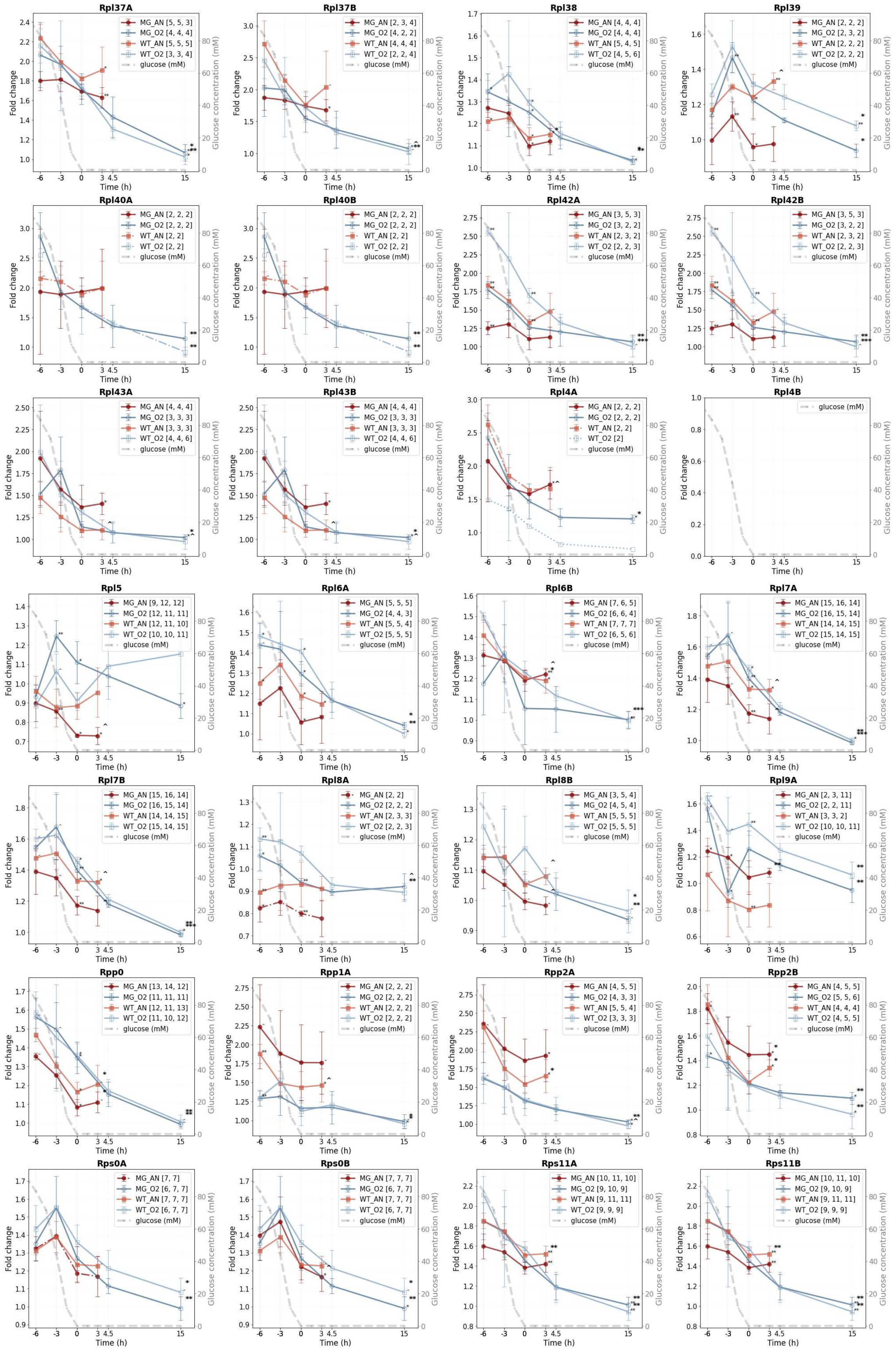
Rpl37A
MG_AN [5, 5, 3]
MG_O2 [4, 4, 4]
WT_AN [5, 5, 5]
WT_O2 [3, 3, 4]
glucose (mM)
Rpl37B
MG_AN [2, 3, 4]
MG_O2 [4, 2, 2]
WT_AN [4, 4, 4]
WT_O2 [2, 2, 4]
glucose (mM)
Rpl38
MG_AN [4, 4, 4]
MG_O2 [4, 4, 4]
WT_AN [5, 4, 5]
WT_O2 [4, 5, 6]
glucose (mM)
Rpl39
MG_AN [2, 2, 2]
MG_O2 [2, 3, 2]
WT_AN [2, 2, 2]
WT_O2 [2, 2, 2]
glucose (mM)
Rpl40A
MG_AN [2, 2, 2]
MG_O2 [2, 2, 2]
WT_AN [2, 2]
WT_O2 [2, 2]
glucose (mM)
Rpl40B
MG_AN [2, 2, 2]
MG_O2 [2, 2, 2]
WT_AN [2, 2]
WT_O2 [2, 2]
glucose (mM)
Rpl42A
MG_AN [3, 5, 3]
MG_O2 [3, 2, 2]
WT_AN [2, 3, 2]
WT_O2 [2, 2, 3]
glucose (mM)
Rpl42B
MG_AN [3, 5, 3]
MG_O2 [3, 2, 2]
WT_AN [2, 3, 2]
WT_O2 [2, 2, 3]
glucose (mM)
Rpl43A
MG_AN [4, 4, 4]
MG_O2 [3, 3, 3]
WT_AN [3, 3, 3]
WT_O2 [4, 4, 6]
glucose (mM)
Rpl43B
MG_AN [4, 4, 4]
MG_O2 [3, 3, 3]
WT_AN [3, 3, 3]
WT_O2 [4, 4, 6]
glucose (mM)
Rpl4A
MG_AN [2, 2, 2]
MG_O2 [2, 2, 2]
WT_AN [2, 2]
WT_O2 [2]
glucose (mM)
Rpl4B
glucose (mM)
Rpl5
MG_AN [9, 12, 12]
MG_O2 [12, 11, 11]
WT_AN [12, 11, 10]
WT_O2 [10, 10, 11]
glucose (mM)
Rpl6A
MG_AN [5, 5, 5]
MG_O2 [4, 4, 3]
WT_AN [5, 5, 4]
WT_O2 [5, 5, 5]
glucose (mM)
Rpl6B
MG_AN [7, 6, 5]
MG_O2 [6, 6, 4]
WT_AN [7, 7, 7]
WT_O2 [6, 5, 6]
glucose (mM)
Rpl7A
MG_AN [15, 16, 14]
MG_O2 [16, 15, 14]
WT_AN [14, 14, 15]
WT_O2 [15, 14, 15]
glucose (mM)
Rpl7B
MG_AN [15, 16, 14]
MG_O2 [16, 15, 14]
WT_AN [14, 14, 15]
WT_O2 [15, 14, 15]
glucose (mM)
Rpl8A
MG_AN [2, 2]
MG_O2 [2, 2, 2]
WT_AN [2, 3, 3]
WT_O2 [2, 2, 3]
glucose (mM)
Rpl8B
MG_AN [3, 5, 4]
MG_O2 [4, 5, 4]
WT_AN [5, 5, 5]
WT_O2 [5, 5, 5]
glucose (mM)
Rpl9A
MG_AN [2, 3, 11]
MG_O2 [2, 2, 11]
WT_AN [3, 3, 2]
WT_O2 [10, 10, 11]
glucose (mM)
Rpp0
MG_AN [13, 14, 12]
MG_O2 [11, 11, 11]
WT_AN [12, 11, 13]
WT_O2 [11, 10, 12]
glucose (mM)
Rpp1A
MG_AN [2, 2, 2]
MG_O2 [2, 2, 2]
WT_AN [2, 2, 2]
WT_O2 [2, 2, 2]
glucose (mM)
Rpp2A
MG_AN [4, 5, 5]
MG_O2 [4, 3, 3]
WT_AN [5, 5, 4]
WT_O2 [3, 3, 3]
glucose (mM)
Rpp2B
MG_AN [4, 5, 5]
MG_O2 [5, 5, 6]
WT_AN [4, 4, 4]
WT_O2 [4, 5, 5]
glucose (mM)
Rps0A
MG_AN [7, 7]
MG_O2 [6, 7, 7]
WT_AN [7, 7, 7]
WT_O2 [6, 7, 7]
glucose (mM)
Rps0B
MG_AN [7, 7, 7]
MG_O2 [6, 7, 7]
WT_AN [7, 7, 7]
WT_O2 [6, 7, 7]
glucose (mM)
Rps11A
MG_AN [10, 11, 10]
MG_O2 [9, 10, 9]
WT_AN [9, 11, 11]
WT_O2 [9, 9, 9]
glucose (mM)
Rps11B
MG_AN [10, 11, 10]
MG_O2 [9, 10, 9]
WT_AN [9, 11, 11]
WT_O2 [9, 9, 9]
glucose (mM)
Fold change
Glucose concentration (mM)
Time (h)

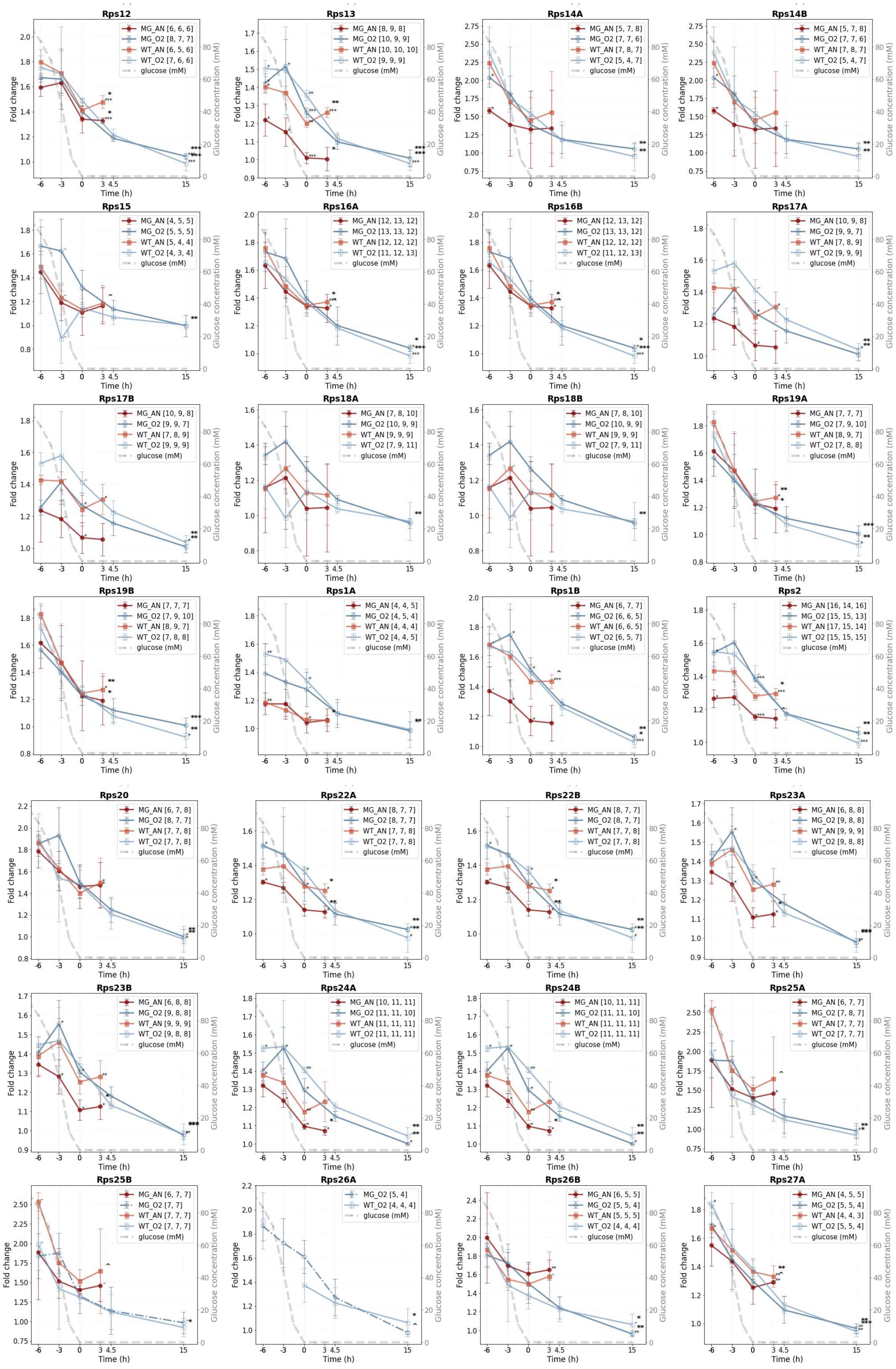
Rps12
MG_AN [6, 6, 6]
MG_O2 [8, 7, 7]
WT_AN [6, 5, 6]
WT_O2 [7, 6, 6]
glucose (mM)
Rps13
MG_AN [8, 9, 8]
MG_O2 [10, 9, 9]
WT_AN [10, 10, 10]
WT_O2 [9, 9, 9]
glucose (mM)
Rps14A
MG_AN [5, 7, 8]
MG_O2 [7, 7, 6]
WT_AN [7, 8, 7]
WT_O2 [5, 4, 7]
glucose (mM)
Rps14B
MG_AN [5, 7, 8]
MG_O2 [7, 7, 6]
WT_AN [7, 8, 7]
WT_O2 [5, 4, 7]
glucose (mM)
Rps15
MG_AN [4, 5, 5]
MG_O2 [5, 5, 5]
WT_AN [5, 4, 4]
WT_O2 [4, 3, 4]
glucose (mM)
Rps16A
MG_AN [12, 13, 12]
MG_O2 [13, 13, 12]
WT_AN [12, 12, 12]
WT_O2 [11, 12, 13]
glucose (mM)
Rps16B
MG_AN [12, 13, 12]
MG_O2 [13, 13, 12]
WT_AN [12, 12, 12]
WT_O2 [11, 12, 13]
glucose (mM)
Rps17A
MG_AN [10, 9, 8]
MG_O2 [9, 9, 7]
WT_AN [7, 8, 9]
WT_O2 [9, 9, 9]
glucose (mM)
Rps17B
MG_AN [10, 9, 8]
MG_O2 [9, 9, 7]
WT_AN [7, 8, 9]
WT_O2 [9, 9, 9]
glucose (mM)
Rps18A
MG_AN [7, 8, 10]
MG_O2 [10, 9, 9]
WT_AN [9, 9, 9]
WT_O2 [7, 9, 11]
glucose (mM)
Rps18B
MG_AN [7, 8, 10]
MG_O2 [10, 9, 9]
WT_AN [9, 9, 9]
WT_O2 [7, 9, 11]
glucose (mM)
Rps19A
MG_AN [7, 7, 7]
MG_O2 [7, 9, 10]
WT_AN [8, 9, 7]
WT_O2 [7, 8, 8]
glucose (mM)
Rps19B
MG_AN [7, 7, 7]
MG_O2 [7, 9, 10]
WT_AN [8, 9, 7]
WT_O2 [7, 8, 8]
glucose (mM)
Rps1A
MG_AN [4, 4, 5]
MG_O2 [4, 5, 4]
WT_AN [4, 4, 4]
WT_O2 [4, 4, 5]
glucose (mM)
Rps1B
MG_AN [6, 7, 7]
MG_O2 [6, 6, 5]
WT_AN [6, 6, 5]
WT_O2 [6, 5, 7]
glucose (mM)
Rps2
MG_AN [16, 14, 16]
MG_O2 [15, 15, 13]
WT_AN [17, 15, 14]
WT_O2 [15, 15, 15]
glucose (mM)
Rps20
MG_AN [6, 7, 8]
MG_O2 [8, 7, 7]
WT_AN [7, 7, 8]
WT_O2 [7, 7, 8]
glucose (mM)
Rps22A
MG_AN [8, 7, 7]
MG_O2 [8, 7, 7]
WT_AN [7, 7, 8]
WT_O2 [7, 7, 8]
glucose (mM)
Rps22B
MG_AN [8, 7, 7]
MG_O2 [8, 7, 7]
WT_AN [7, 7, 8]
WT_O2 [7, 7, 8]
glucose (mM)
Rps23A
MG_AN [6, 8, 8]
MG_O2 [9, 8, 8]
WT_AN [9, 9, 9]
WT_O2 [9, 8, 8]
glucose (mM)
Rps23B
MG_AN [6, 8, 8]
MG_O2 [9, 8, 8]
WT_AN [9, 9, 9]
WT_O2 [9, 8, 8]
glucose (mM)
Rps24A
MG_AN [10, 11, 11]
MG_O2 [11, 11, 10]
WT_AN [11, 11, 11]
WT_O2 [11, 11, 11]
glucose (mM)
Rps24B
MG_AN [10, 11, 11]
MG_O2 [11, 11, 10]
WT_AN [11, 11, 11]
WT_O2 [11, 11, 11]
glucose (mM)
Rps25A
MG_AN [6, 7, 7]
MG_O2 [7, 8, 7]
WT_AN [7, 7, 7]
WT_O2 [7, 7, 7]
glucose (mM)
Rps25B
MG_AN [6, 7, 7]
MG_O2 [7, 7]
WT_AN [7, 7, 7]
WT_O2 [7, 7, 7]
glucose (mM)
Rps26A
MG_O2 [5, 4]
WT_O2 [4, 4, 4]
glucose (mM)
Rps26B
MG_AN [6, 5, 5]
MG_O2 [5, 5, 4]
WT_AN [5, 5, 5]
WT_O2 [4, 4, 4]
glucose (mM)
Rps27A
MG_AN [4, 5, 5]
MG_O2 [5, 5, 4]
WT_AN [4, 4, 3]
WT_O2 [5, 5, 4]
glucose (mM)
Fold change
Time (h)
Glucose concentration (mM)

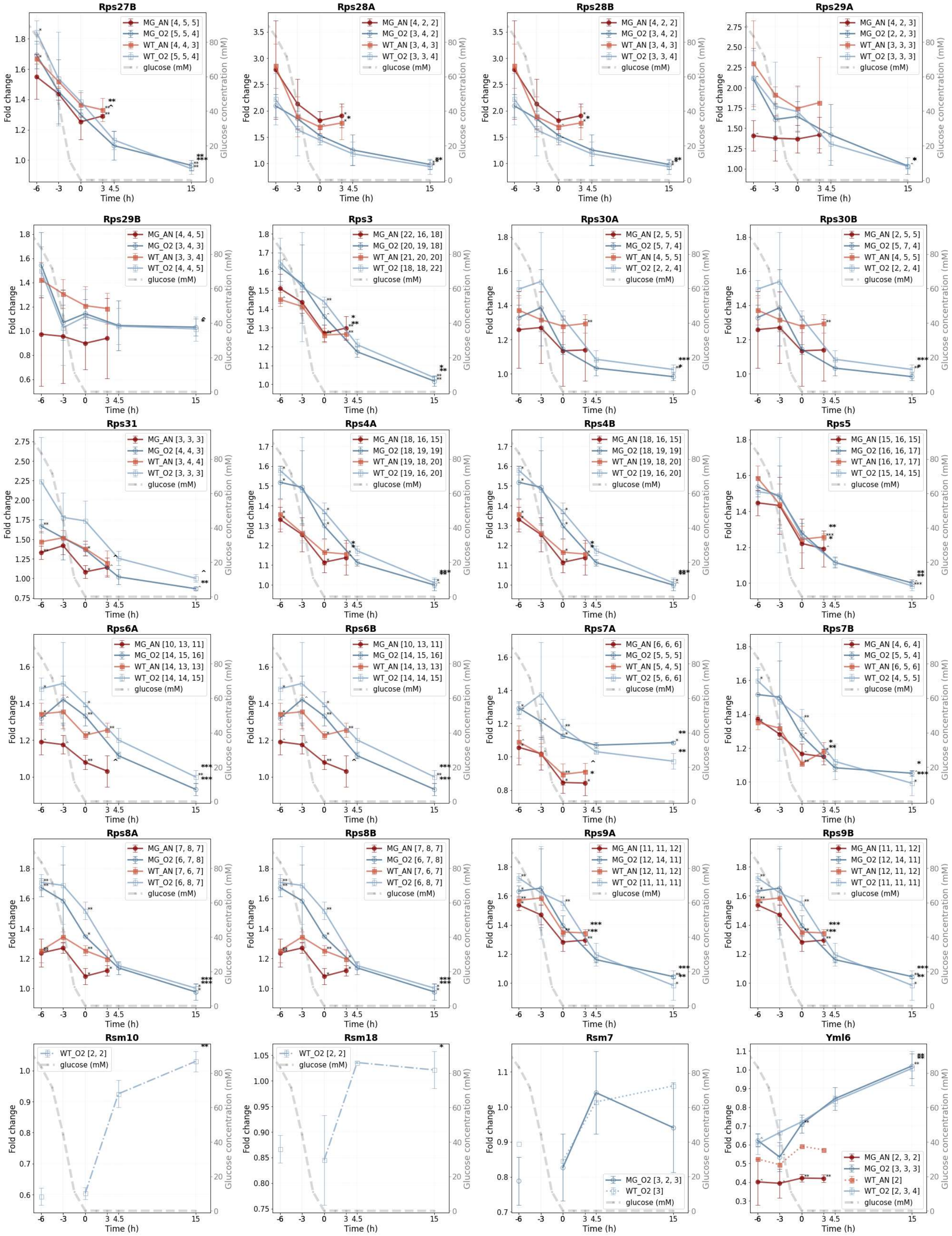

SI Figure 9.6. Fold change line graphs for proteins of selected pathways for the control yeast and the MG strain under aerobic and anaerobic conditions. The biological-replicate-averaged FCs of CEN.PK113-7D and IMX372 (MG) were plotted against the time relative to glucose depletion (0 h) in hours. Red: the MG strain under anaerobic conditions, dark blue: the MG strain under aerobic conditions, orange: the control strain under anaerobic conditions, light blue: the control strain under aerobic conditions. The error bars show the standard deviation of the mean of the three biological replicates. In the legend, the numbers between brackets represent the number of unique peptides that were found in each biological replicate. The grey dashed line represents the glucose concentration over time (mM, secondary y-axis). Asterisks (*) and circumflexes (^) indicate the significance between either the (an)aerobic experiments (black annotation) or between the ME and MS phase. P-value levels are: $p < 0.001$ (***), $p < 0.01$ (**), $p < 0.05$ (*), and $p < 0.1$ (^).

ROS

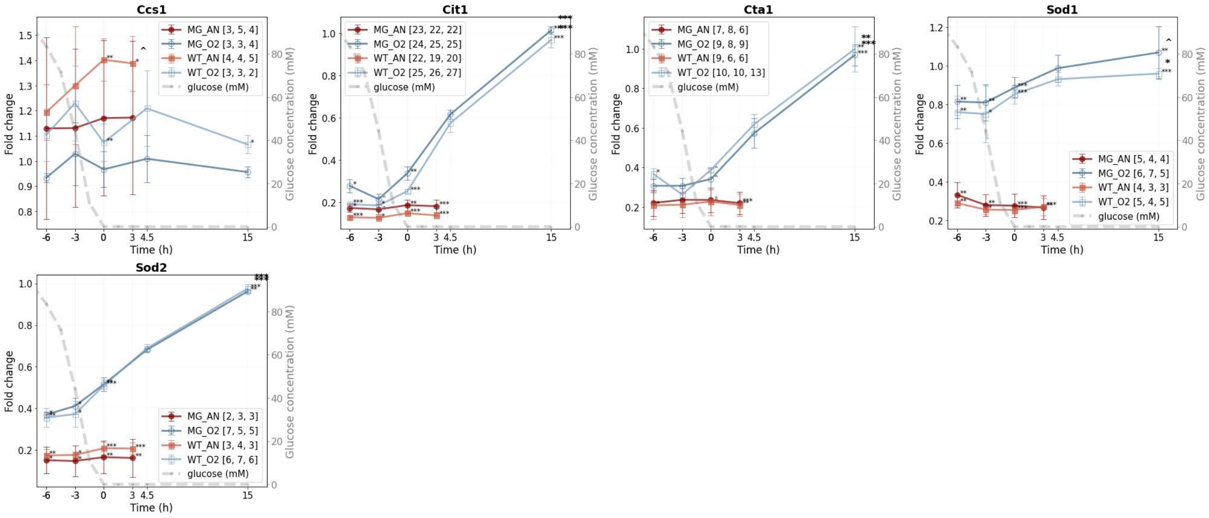

SI Figure 9.7. Fold change line graphs for proteins of selected pathways for the control yeast and the MG strain under aerobic and anaerobic conditions. The biological-replicate-averaged FCs of CEN.PK113-7D and IMX372 (MG) were plotted against the time relative to glucose depletion (0 h) in hours. Red: the MG strain under anaerobic conditions, dark blue: the MG strain under aerobic conditions, orange: the control strain under anaerobic conditions, light blue: the control strain under aerobic conditions. The error bars show the standard deviation of the mean of the three biological replicates. In the legend, the numbers between brackets represent the number of unique peptides that were found in each biological replicate. The grey dashed line represents the glucose concentration over time (mM, secondary y-axis). Asterisks (*) and circumflexes (^) indicate the significance between either the (an)aerobic experiments (black annotation) or between the ME and MS phase. P-value levels are: $p < 0.001$ (***), $p < 0.01$ (**), $p < 0.05$ (*), and $p < 0.1$ (^).

Proteasome

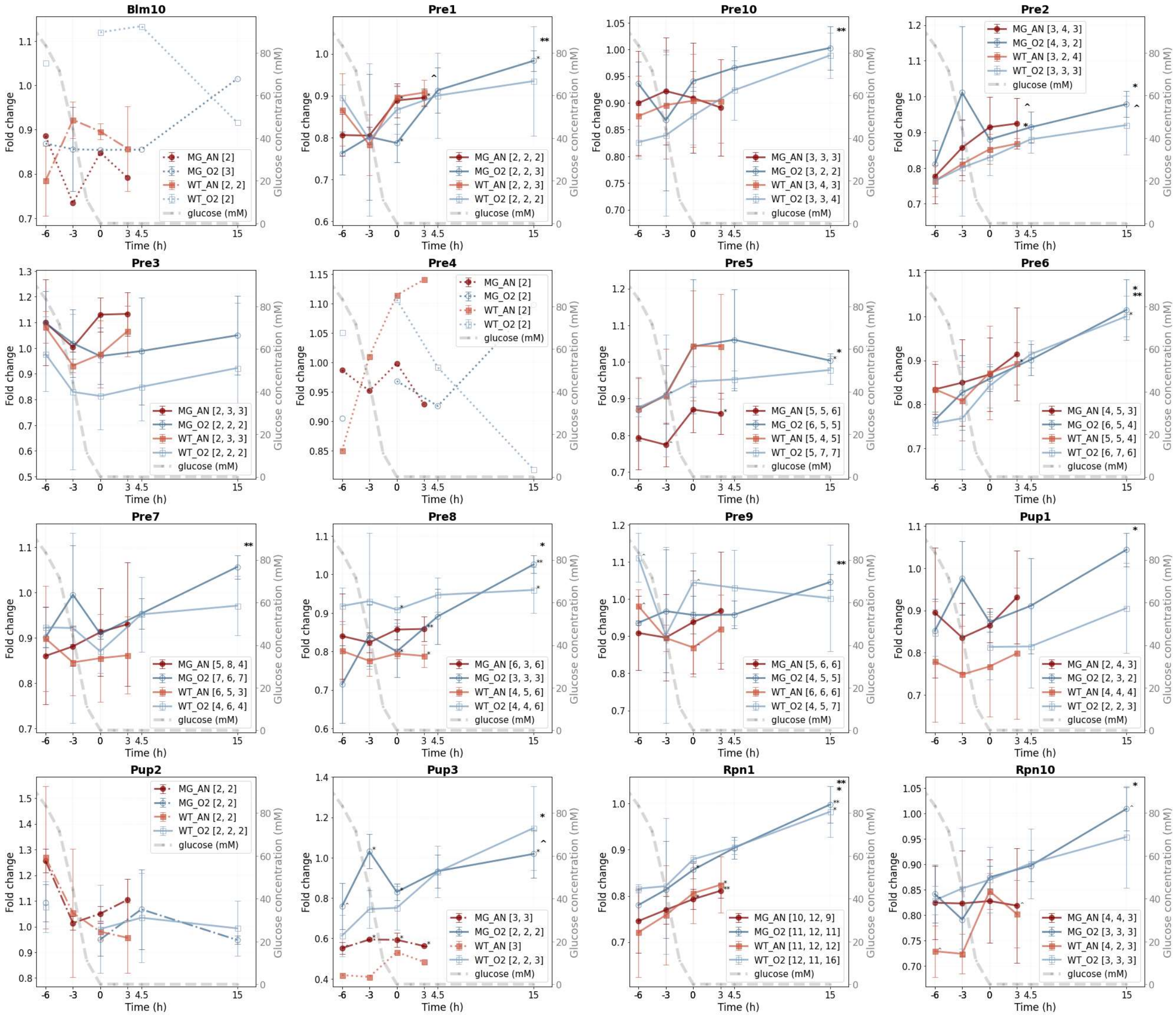
Blm10
MG_AN [2]
MG_O2 [3]
WT_AN [2, 2]
WT_O2 [2]
glucose (mM)
Pre1
MG_AN [2, 2, 2]
MG_O2 [2, 2, 3]
WT_AN [2, 2, 3]
WT_O2 [2, 2, 2]
glucose (mM)
Pre10
MG_AN [3, 3, 3]
MG_O2 [3, 2, 2]
WT_AN [3, 4, 3]
WT_O2 [3, 3, 4]
glucose (mM)
Pre2
MG_AN [3, 4, 3]
MG_O2 [4, 3, 2]
WT_AN [3, 2, 4]
WT_O2 [3, 3, 3]
glucose (mM)
Pre3
MG_AN [2, 3, 3]
MG_O2 [2, 2, 2]
WT_AN [2, 3, 3]
WT_O2 [2, 2, 2]
glucose (mM)
Pre4
MG_AN [2]
MG_O2 [2]
WT_AN [2]
WT_O2 [2]
glucose (mM)
Pre5
MG_AN [5, 5, 6]
MG_O2 [6, 5, 5]
WT_AN [5, 4, 5]
WT_O2 [5, 7, 7]
glucose (mM)
Pre6
MG_AN [4, 5, 3]
MG_O2 [6, 5, 4]
WT_AN [5, 5, 4]
WT_O2 [6, 7, 6]
glucose (mM)
Pre7
MG_AN [5, 8, 4]
MG_O2 [7, 6, 7]
WT_AN [6, 5, 3]
WT_O2 [4, 6, 4]
glucose (mM)
Pre8
MG_AN [6, 3, 6]
MG_O2 [3, 3, 3]
WT_AN [4, 5, 6]
WT_O2 [4, 4, 6]
glucose (mM)
Pre9
MG_AN [5, 6, 6]
MG_O2 [4, 5, 5]
WT_AN [6, 6, 6]
WT_O2 [4, 5, 7]
glucose (mM)
Pup1
MG_AN [2, 4, 3]
MG_O2 [2, 3, 2]
WT_AN [4, 4, 4]
WT_O2 [2, 2, 3]
glucose (mM)
Pup2
MG_AN [2, 2]
MG_O2 [2, 2]
WT_AN [2, 2]
WT_O2 [2, 2, 2]
glucose (mM)
Pup3
MG_AN [3, 3]
MG_O2 [2, 2, 2]
WT_AN [3]
WT_O2 [2, 2, 3]
glucose (mM)
Rpn1
MG_AN [10, 12, 9]
MG_O2 [11, 12, 11]
WT_AN [11, 12, 12]
WT_O2 [12, 11, 16]
glucose (mM)
Rpn10
MG_AN [4, 4, 3]
MG_O2 [3, 3, 3]
WT_AN [4, 2, 3]
WT_O2 [3, 3, 3]
glucose (mM)
Fold change
Glucose concentration (mM)
Time (h)

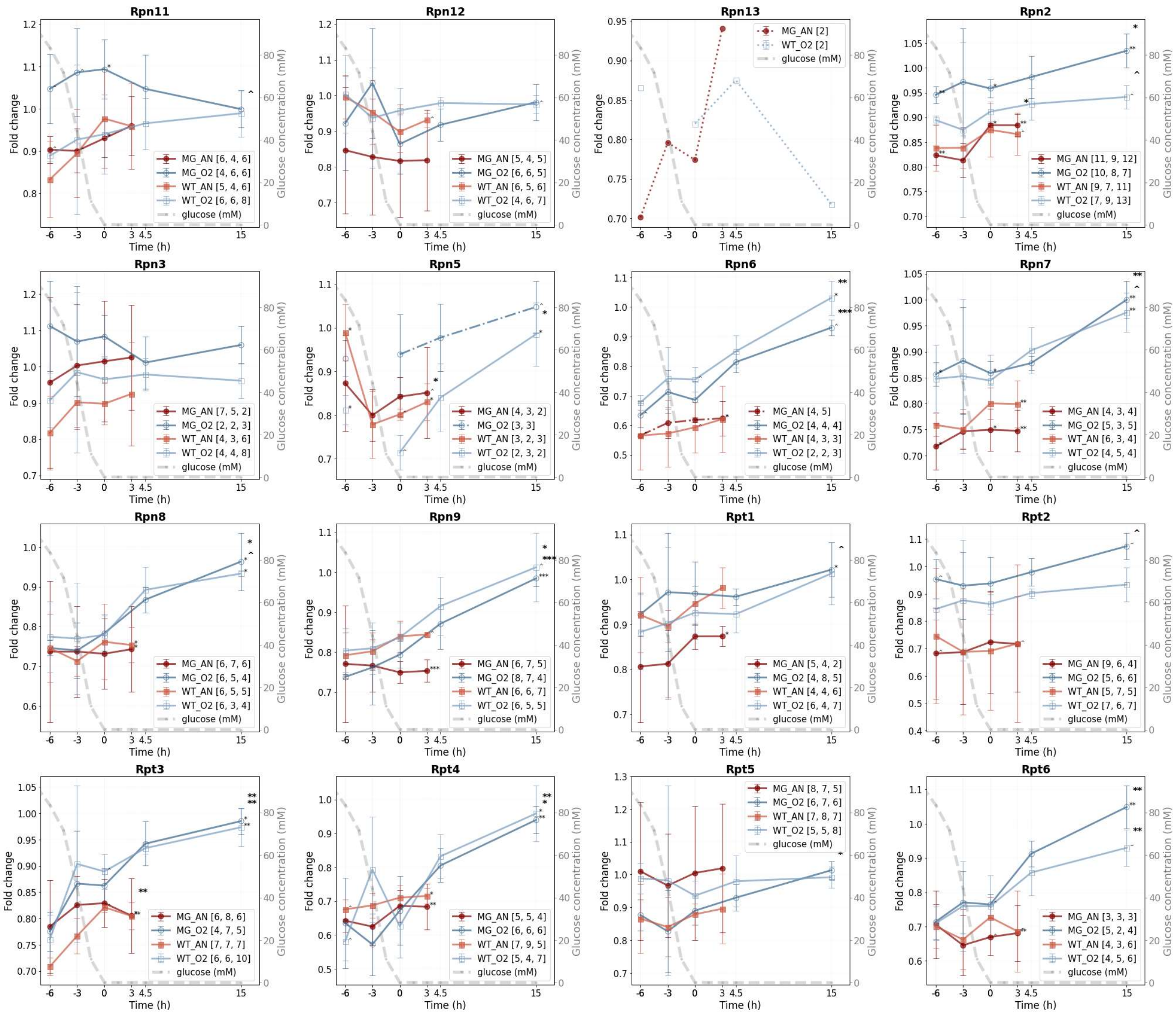

SI Figure 9.8. Fold change line graphs for proteins of selected pathways for the control yeast and the MG strain under aerobic and anaerobic conditions. The biological-replicate-averaged FCs of CEN.PK113-7D and IMX372 (MG) were plotted against the time relative to glucose depletion (0 h) in hours. Red: the MG strain under anaerobic conditions, dark blue: the MG strain under aerobic conditions, orange: the control strain under anaerobic conditions, light blue: the control strain under aerobic conditions. The error bars show the standard deviation of the mean of the three biological replicates. In the legend, the numbers between brackets represent the number of unique peptides that were found in each biological replicate. The grey dashed line represents the glucose concentration over time (mM, secondary y-axis). Asterisks (*) and circumflexes (^) indicate the significance between either the (an)aerobic experiments (black annotation) or between the ME and MS phase. P-value levels are: $p < 0.001$ (***), $p < 0.01$ (**), $p < 0.05$ (*), and $p < 0.1$ (^).

Protein degradation 26S proteasome complex

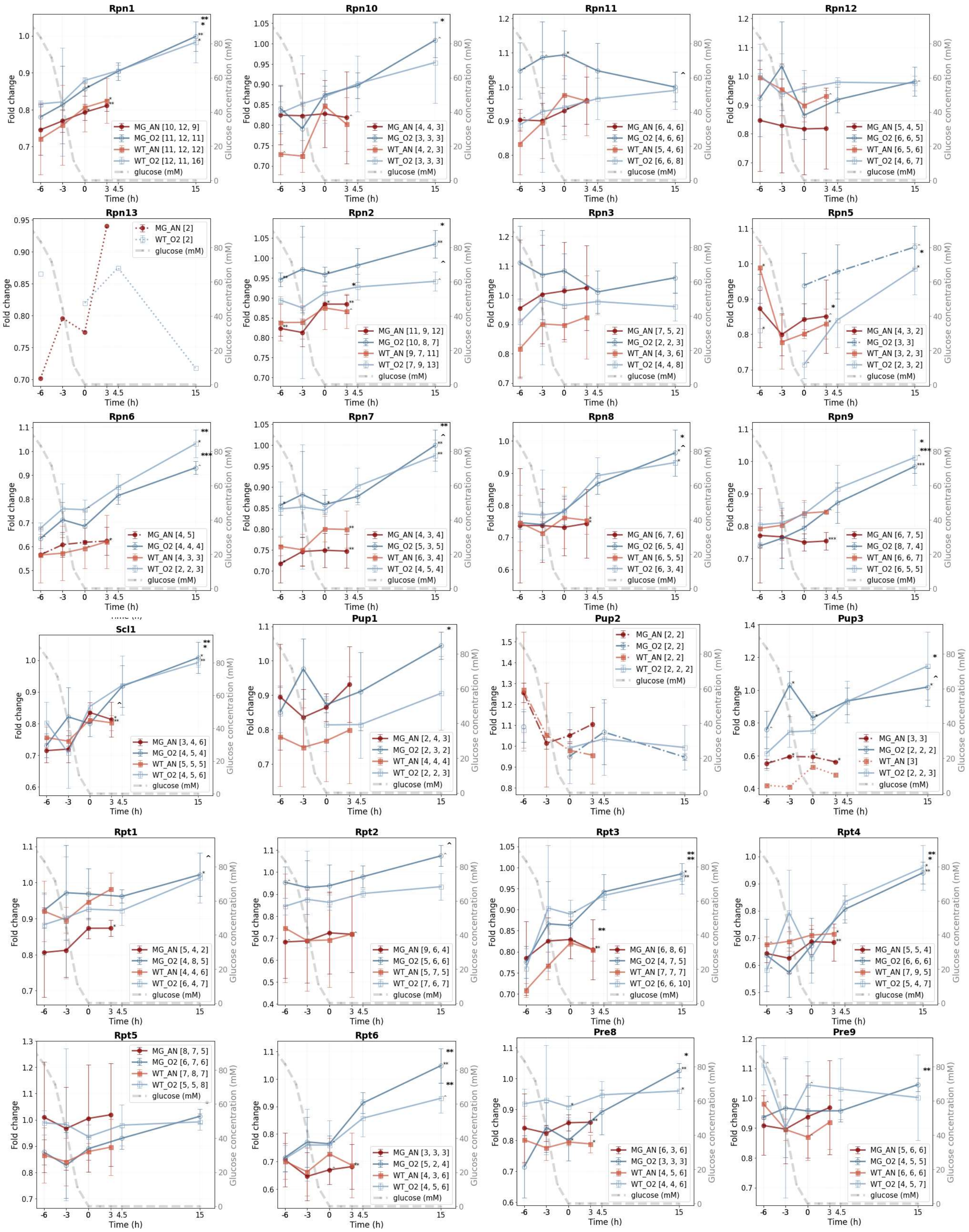

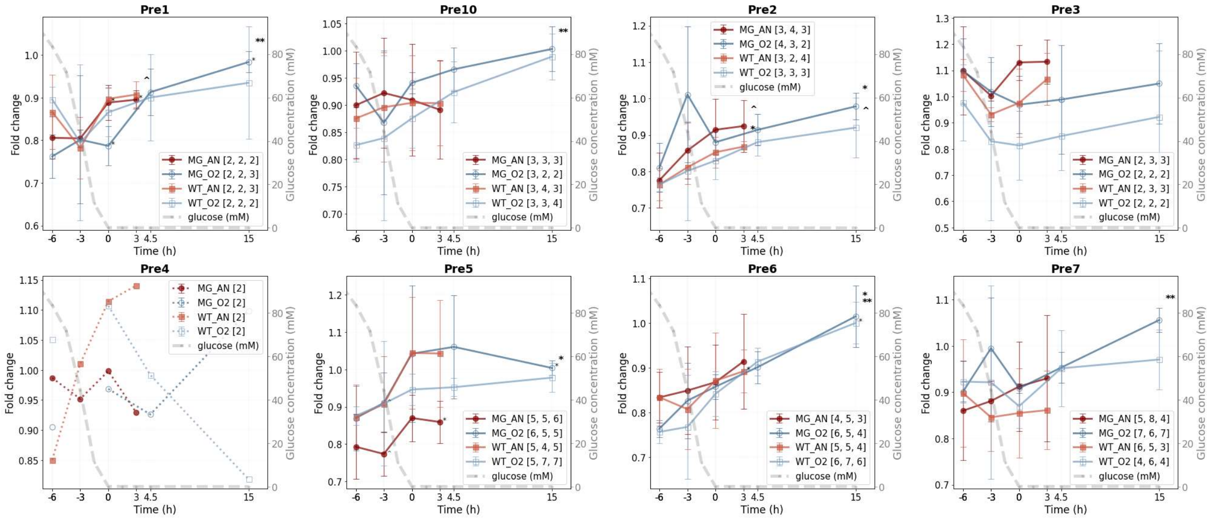

SI Figure 9.9. Fold change line graphs for proteins of selected pathways for the control yeast and the MG strain under aerobic and anaerobic conditions. The biological-replicate-averaged FCs of CEN.PK113-7D and IMX372 (MG) were plotted against the time relative to glucose depletion (0 h) in hours. Red: the MG strain under anaerobic conditions, dark blue: the MG strain under aerobic conditions, orange: the control strain under anaerobic conditions, light blue: the control strain under aerobic conditions. The error bars show the standard deviation of the mean of the three biological replicates. In the legend, the numbers between brackets represent the number of unique peptides that were found in each biological replicate. The grey dashed line represents the glucose concentration over time (mM, secondary y-axis). Asterisks (*) and circumflexes (^) indicate the significance between either the (an)aerobic experiments (black annotation) or between the ME and MS phase. P-value levels are: $p < 0.001$ (***), $p < 0.01$ (**), $p < 0.05$ (*), and $p < 0.1$ (^).
